# *Panax quinquefolius*-derived nanovesicles: a synergistic nanotherapy for postmenopausal osteoporosis via modulating gut microbiota, MYC-mediated inflammation, and Nrf2/HO-1 antioxidant pathway

**DOI:** 10.3389/fmicb.2026.1836521

**Published:** 2026-07-22

**Authors:** Qin Yin, Jun Gu, Ruijun Bai, Kai Zhao, Qiubo Wang, Yanbin Liu, Yu Liu, Haifeng Li

**Affiliations:** Department of Orthopedics, Wuxi Ninth People's Hospital Affiliated to Soochow University, Wuxi, Jiangsu, China

**Keywords:** gut microbiota, gut-bone axis, MYC pathway, nanovesicles, Nrf2/HO-1, osteoporosis, oxidative stress, *Panax quinquefolius*

## Abstract

**Background:**

Plant-derived nanovesicles (NVs) have emerged as promising natural nanotherapeutics for metabolic and inflammatory diseases due to their inherent biocompatibility, multi-target potential, and ability to mediate cross-kingdom communication. Postmenopausal osteoporosis (PMOP), a prevalent skeletal disorder driven by estrogen deficiency, is characterized by gut-bone axis dysregulation, chronic inflammation, and excessive oxidative stress—pathological features that demand synergistic multi-target interventions. This study aimed to characterize *Panax quinquefolius*-derived nanovesicles (PQs) and systematically validate their anti-osteoporotic efficacy, bone-targeting property, and underlying molecular mechanisms in preclinical models.

**Methods:**

PQs were isolated via modified differential centrifugation and characterized using transmission electron microscopy (TEM), nanoparticle tracking analysis (NTA), and high-performance liquid chromatography (HPLC). Their bone-targeting capacity was evaluated via *in vivo* biodistribution and cellular uptake assays. Anti-osteoporotic efficacy was assessed in ovariectomized (OVX) mice using dual-energy X-ray absorptiometry (DXA), micro-computed tomography (μCT), and histomorphometric analysis. Gut microbiota modulation was analyzed via 16S rRNA gene sequencing. Network pharmacology, molecular docking, and loss-of-function experiments (pharmacological inhibition and siRNA-mediated Nrf2 silencing) were employed to elucidate the core mechanisms.

**Results:**

PQs exhibited a typical cup-shaped morphology (mean diameter ~89 nm), negative zeta potential (−42.21 ± 0.12 mV), and stable batch-to-batch naringin content (0.65 ± 0.03 μg/mL). *In vivo* studies confirmed intrinsic bone-targeting of PQs, with >35% uptake by mouse bone marrow mesenchymal stem cells (mBMSCs) at 48 h post-injection. In OVX mice, PQs dose-dependently improved bone mineral density (BMD), trabecular microarchitecture (increased BV/TV, Tb. N, Tb. Th; decreased Tb. Sp), and serum osteogenic markers (ALP, OCN, Ca), while reducing osteoclastic markers (TRAP5b, CTX-I) and systemic inflammation (IL-6, KC, LPS). PQs treatment was associated with normalization Firmicutes/Bacteroidetes ratio, enriching probiotics (Bifidobacterium, Lactobacillus), and suppressing pathogenic bacteria (Desulfovibrio, Enterococcus). Mechanistically, PQs exerted therapeutic effects by synergistically suppressing MYC-mediated inflammatory signaling and activating the Nrf2/HO-1 antioxidant pathway. Pharmacological inhibition (ML385 for Nrf2, HY-11798 for HO-1) or genetic silencing of Nrf2 completely abolished the osteoprotective, anti-inflammatory, and antioxidant effects of PQs.

**Conclusion:**

*Panax quinquefolius*-derived nanovesicles represent a novel synergistic nanotherapy for PMOP. Their therapeutic effects are mediated by concurrent modulation of gut microbiota homeostasis, suppression of MYC-dependent inflammation, and activation of the Nrf2/HO-1 pathway. Given their natural origin, biocompatibility, and multi-target action, PQs hold significant potential for clinical translation as a safe and effective alternative for PMOP management.

## Graphical abstract

**Figure:**
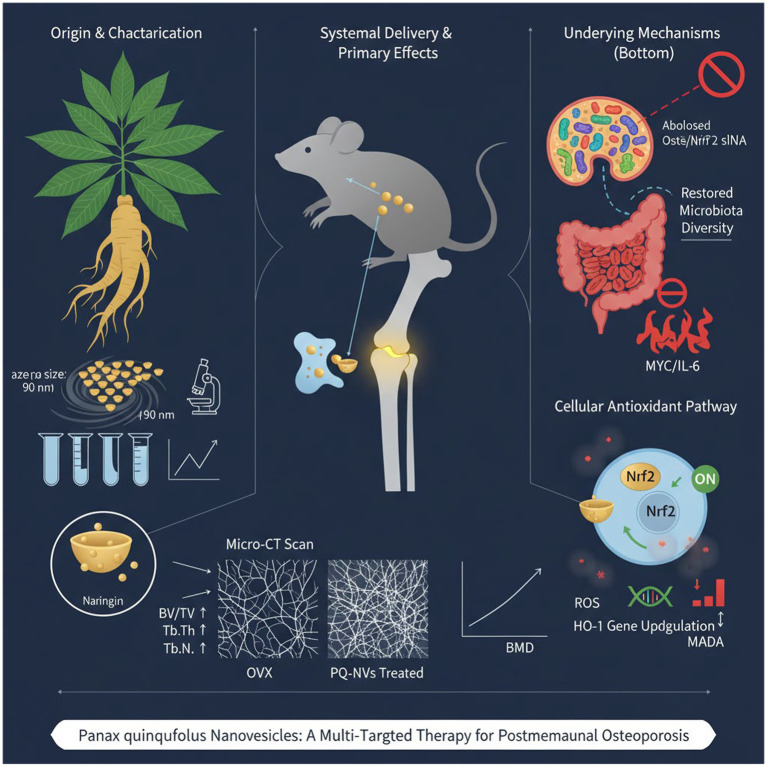
*Panax quinquefolius*-derived nanovesicles (PQs) alleviate postmenopausal osteoporosis through multi-targeted mechanisms. Systemically administered PQs exhibit bone-targeting properties, leading to improved bone mineral density and microstructure in ovariectomized mice. The therapeutic effects are mediated by restoring gut microbiota diversity, suppressing systemic inflammation via the MYC/IL-6 pathway, and activating the cellular Nrf2/HO-1 antioxidant defense system. Genetic or pharmacological inhibition of Nrf2 abolishes the osteoprotective benefits, confirming its central role.

## Introduction

Osteoporosis, a skeletal disorder hallmarked by progressive bone loss and elevated fracture risk, represents a major and growing global health challenge (Arceo-Mendoza and Camacho, 2021; Baccaro et al., 2015). Its impact is substantial, affecting over 200 million individuals worldwide; the associated fractures lead to significant morbidity, diminished quality of life, and considerable healthcare costs (Brown, 2021). The pathogenesis of this disease is multifactorial, arising from a complex interplay of hormonal changes, nutritional status, and lifestyle factors (Fischer and Haffner-Luntzer, 2022; Li et al., 2020). In postmenopausal women, the age-related decline in ovarian function and the consequent drop in estrogen levels serve as a primary accelerator of bone deterioration (Xu et al., 2022). This estrogen deficiency fundamentally disrupts the bone remodeling process—a delicate balance between formation and resorption—by suppressing osteoblast activity while simultaneously enhancing osteoclast function (Muñoz et al., 2020; Silverstein et al., 2024; Yao et al., 2025).

Beyond this hormonal axis, recent evidence underscores the crucial roles of chronic low-grade inflammation and oxidative stress in driving disease progression. The NLRP3 inflammasome, a pivotal regulator of innate immune responses, is a central node in this inflammatory cascade: its activation triggers the maturation and secretion of pro-inflammatory cytokines including IL-1β and IL-18, which directly promote osteoclastogenesis and bone resorption in the skeletal microenvironment. Notably, the origin of this persistent systemic inflammation is increasingly linked to intestinal dysbiosis, establishing the gut-bone axis as a critical regulatory pathway in bone metabolism. The gut microbiota, a complex microbial community colonizing the gastrointestinal tract, plays an indispensable role in maintaining bone homeostasis by regulating systemic immunity, nutrient absorption, and inflammatory responses. Dysbiosis—an imbalance in the composition and diversity of the gut microbiota—impairs intestinal barrier integrity, leading to increased intestinal permeability (so-called “leaky gut”; Guan et al., 2023b). This permeability allows microbial metabolites and pathogens to translocate into the circulation, initiating and perpetuating systemic low-grade inflammation, which in turn exacerbates osteoclast-mediated bone loss. Targeting the gut-bone axis to restore microbial homeostasis and alleviate inflammation thus represents a promising multi-targeted therapeutic strategy for PMOP.

Indeed, research into the gut-bone axis has intensified in recent years, revealing how the complex ecological composition of gut bacteria influences bone homeostasis and host immune responses (Guan et al., 2023b; Guan et al., 2023a; Guan et al., 2022; Guan et al., 2025b; Zheng et al., 2025; Chen et al., 2024). Dysbiosis-induced intestinal barrier dysfunction is now recognized as a major contributor to the chronic inflammatory state underlying osteoporosis, with microbial disturbances directly activating the NLRP3 inflammasome in the systemic circulation (Maagensen et al., 2023). The subsequent secretion of IL-1β and IL-18 further drives osteoclast development and accelerates bone resorption, reinforcing the rationale for multi-targeted therapeutic strategies that simultaneously restore gut microbiota balance and dampen NLRP3-mediated inflammatory signaling in PMOP (Zaiss et al., 2019; Zhang et al., 2024).

*Panax quinquefolius* (American ginseng), a well-documented medicinal herb in traditional Chinese and Western medicine, has long been valued for its diverse pharmacological activities (Mancuso and Santangelo, 2017; Xia et al., 2024), including anti-inflammation, immunomodulation, antioxidant effects, and prebiotic-like properties that regulate gut microbiota composition (Ghosh et al., 2020). Preclinical studies have demonstrated the potential of *Panax quinquefolius* in ameliorating various metabolic disorders, with its therapeutic effects closely associated with the modulation of the gut microbiota and subsequent attenuation of systemic inflammation. A growing body of research has revealed that plants exert biological effects in part through the release of nanovesicles (NVs)—nanoscale membrane-bound structures that mediate the intercellular transfer of bioactive molecules such as nucleic acids, proteins, flavonoids, and polysaccharides (Mougenot et al., 2022; Piening and Wachs, 2023; Wadhwa et al., 2019). Moreover, plant-derived NVs have been shown to modulate immune responses, regulate microbial metabolism, and facilitate cross-kingdom communication, making them attractive candidates for the development of novel nanotherapies for inflammatory and metabolic diseases (Huang et al., 2023). Despite these promising attributes, the specific role of *Panax quinquefolius*-derived nanovesicles (PQs) in the prevention and treatment of PMOP, as well as their underlying mechanisms of action—particularly their effects on the gut-bone axis, inflammatory signaling, and oxidative stress—remain largely unexplored (Dad et al., 2021).

Building on these observations, we hypothesized that PQs could mitigate estrogen deficiency-induced bone loss in PMOP through a multi-targeted mechanism involving the modulation of gut microbiota homeostasis, suppression of systemic inflammatory signaling, and activation of cellular antioxidant defense pathways to alleviate oxidative stress in bone tissue. In the present study, we isolated and characterized PQs from *Panax quinquefolius*, and evaluated their anti-osteoporotic efficacy using an established ovariectomized (OVX) mouse model of PMOP—the gold standard preclinical model for studying postmenopausal bone loss. We further elucidated the underlying molecular mechanisms, with a specific focus on the regulatory effects of PQs on the gut-bone axis, MYC-mediated inflammatory signaling, and the Nrf2/HO-1 antioxidant pathway. Our findings aim to provide a novel scientific basis for the development of PQs as a natural, safe, and multi-targeted nanotherapy for the clinical management of PMOP.

## Materials and methods

This study was approved by the Institutional Review Board (IRB) and the Ethics Committee of Wuxi Ninth People’s Hospital Affiliated to Soochow University. All animal experiments were conducted in strict compliance with the ARRIVE guidelines, the U. K. Animals (Scientific Procedures) Act 1986, EU Directive 2010/63/EU, and the National Research Council’s Guide for the Care and Use of Laboratory Animals.

### Isolation and preparation of *Panax quinquefolius*-derived nanovesicles (PQs)

Dried roots of *Panax quinquefolius* (American ginseng) were purchased from a certified medicinal herb supplier (Jiangsu, China) and authenticated by the Department of Pharmacognosy, Yangzhou University. Water-soluble polysaccharide-associated PQs were isolated via a modified differential centrifugation method. Briefly, 500 g of dried *Panax quinquefolius* roots were cut into 1–2 cm sections, suspended in sterile distilled water (1,10, w/v), and subjected to hot water extraction at 100 °C for 4 h with constant stirring. The extract was filtered through four layers of gauze to remove insoluble residues, and the filter residue was re-extracted twice under the same conditions to maximize yield. The combined filtrates were concentrated to a volume of 100 mL using a rotary evaporator (RE-52AA, Yarong, China) at 50 °C under reduced pressure, followed by ethanol precipitation (final ethanol concentration 80%, v/v) at 4 °C for 12 h. The resulting precipitate was collected by centrifugation (8,000 × g, 20 min, 4 °C) and redissolved in sterile distilled water; the ethanol precipitation step was repeated twice for further purification (Ren et al., 2022).

Proteins in the crude extract were removed using the Sevag reagent (chloroform: n-butanol = 4:1, v/v) with vigorous shaking for 30 min, followed by centrifugation (5,000 × g, 15 min, 4 °C) to discard the intermediate protein layer; this step was repeated 3–4 times until no visible protein layer was observed. The final polysaccharide-nanovesicle mixture was precipitated with 80% ethanol, and the precipitate was collected and vacuum freeze-dried (FD-1A-50, Boyikang, China) to obtain lyophilized PQs powder. The yield of PQs was calculated as the percentage of lyophilized powder weight relative to the initial dried root weight (w/w). The main components of PQs were quantified via biochemical assays: total carbohydrate content by the phenol-sulfuric acid method, uronic acid content by the m-hydroxybiphenyl method, and protein content by the BCA protein assay kit (Beyotime, China). Monosaccharide composition analysis was performed via high-performance anion-exchange chromatography with pulsed amperometric detection (HPAEC-PAD, Dionex, USA) after acid hydrolysis (2 M TFA, 120 °C, 2 h).

**Figure 1:**
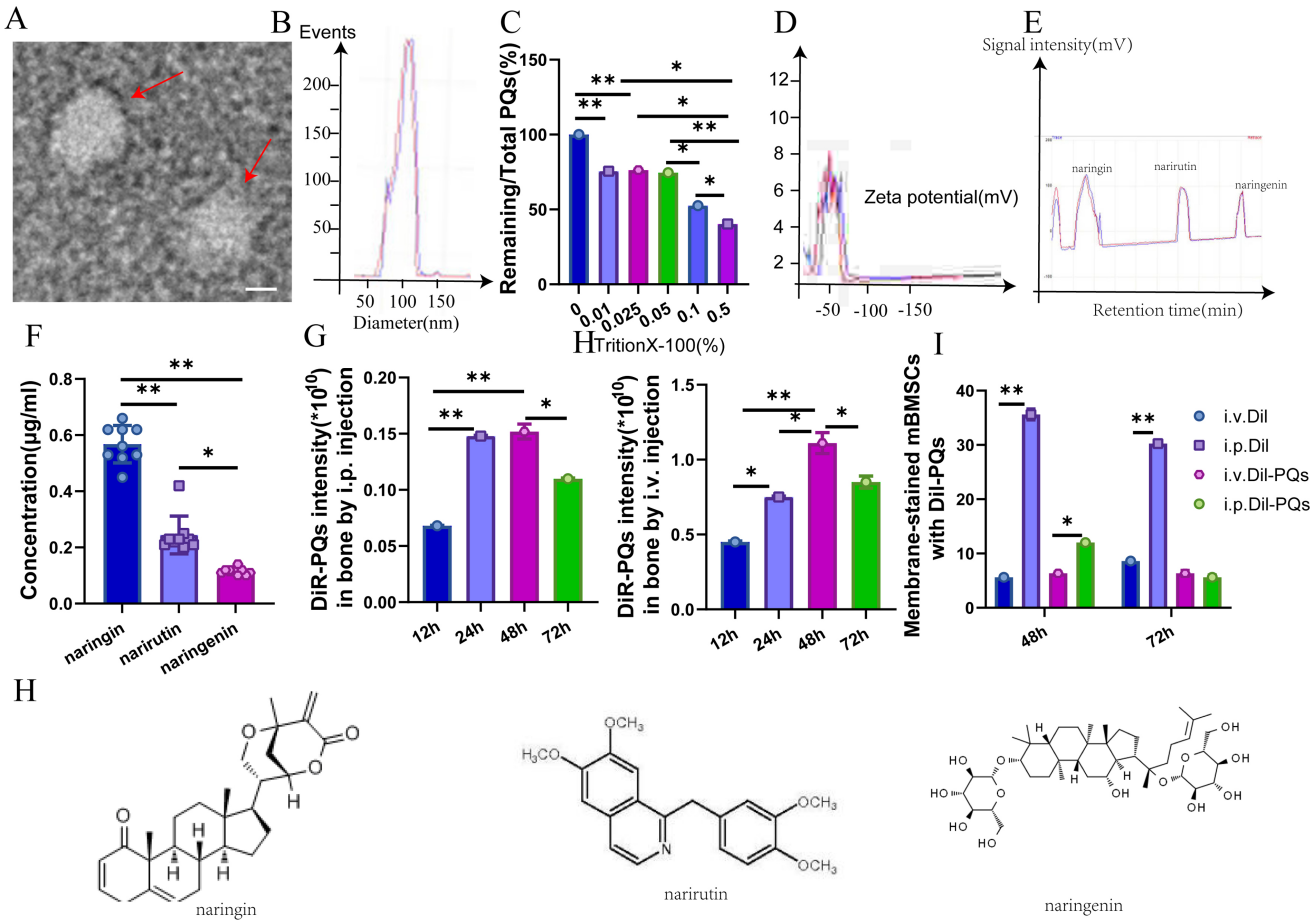
Isolation and physicochemical characterization of *Panax quinquefolius*-derived nanovesicles (PQs). **(A)** Representative transmission electron microscopy (TEM) image showing the cup-shaped morphology of PQs (magnification: ×100,000; scale bar = 50 nm). Red arrows indicate representative cup-shaped nanovesicles with intact lipid bilayer membranes. **(B)** Particle size distribution of PQs as determined by nanoparticle tracking analysis (NTA; detection temperature: 25 °C, particle concentration: 5.92 × 10^11^ particles/mg). **(C)** Membrane integrity assay: PQs were incubated with increasing concentrations of Triton X-100 (0–1.0%, v/v), and particle concentration was quantified by NTA. The dose-dependent decrease in particle concentration confirms the lipid bilayer structure. **(D)** Zeta potential measurement of PQs (*n* = 5 independent batches). **(E)** Representative HPLC chromatogram of PQs at 283 nm. The major peak corresponding to naringin is indicated. ‘DP’ refers to the ‘detected peak’ of the internal standard (uracil). **(F)** Quantitative analysis of naringin concentration across five independent batches (*n* = 5). **(G,H)** Biodistribution of DiR-labeled PQs in major organs (liver, spleen, lung) and femur at 6 h, 12 h, and 24 h after intravenous **(G)** or intraperitoneal **(H)** administration. Fluorescence intensity was quantified using an *in vivo* imaging system (IVIS, PerkinElmer, USA). **(I)** *In vivo* cellular uptake: Flow cytometry analysis (excitation wavelength: 488 nm, emission wavelength: 525 nm) showing the percentage of DiI-labeled PQs internalized by mouse bone marrow mesenchymal stem cells (mBMSCs) isolated from femurs at 48 h post-injection (n=6 mice per group). *p < 0.05, **p < 0.01, ***p < 0.001.

PQs were isolated via a modified differential centrifugation method combined with ethanol precipitation and Sevag reagent deproteinization. Briefly, dried roots of *Panax quinquefolius* (American ginseng) were cut into 1–2 cm sections, suspended in sterile distilled water (1,10, w/v), and subjected to hot water extraction at 100 °C for 4 h with constant stirring. The extract was filtered through four layers of gauze, and the residue was re-extracted twice under the same conditions. The combined filtrates were concentrated to 100 mL using a rotary evaporator at 50 °C under reduced pressure, followed by ethanol precipitation (final ethanol concentration 80%, v/v) at 4 °C for 12 h. The resulting precipitate was collected by centrifugation (8,000 × g, 20 min, 4 °C), redissolved in sterile distilled water, and the ethanol precipitation step was repeated twice. Proteins in the crude extract were removed using the Sevag reagent (chloroform:n-butanol = 4:1, v/v) with vigorous shaking for 30 min, followed by centrifugation (5,000 × g, 15 min, 4 °C) to discard the intermediate protein layer; this step was repeated 3–4 times. The final polysaccharide-nanovesicle mixture was precipitated with 80% ethanol, and the precipitate was collected and vacuum freeze-dried to obtain lyophilized PQs powder. The lyophilized powder was resuspended in sterile water and characterized as nanovesicles by transmission electron microscopy (TEM) and nanoparticle tracking analysis (NTA), confirming the presence of cup-shaped particles with an intact lipid bilayer membrane and a mean diameter of ~89 nm (Figures 1A–C). The yield of PQs was calculated as the percentage of lyophilized powder weight relative to the initial dried root weight (w/w).

### High performance liquid chromatography for quality control of PQs

HPLC was employed for qualitative and quantitative analysis of the key bioactive component (naringin) in PQs and batch-to-batch quality control. Briefly, 0.5 g of lyophilized PQs powder was accurately weighed and dissolved in 10% methanol (v/v) to a final concentration of 10 mg/mL, vortexed for 5 min, and sonicated (40 kHz, 30 min, 25 °C) to facilitate complete extraction of hydrophobic components. The extract was centrifuged (12,000 × g, 10 min, 4 °C), and the supernatant was filtered through a 0.45 μm hydrophilic organic membrane filter (Millipore, USA) prior to HPLC injection.

Analyses were performed on an Agilent 1,200 Series HPLC system (Agilent, USA) equipped with a ZORBAX SB-Aq C18 column (250 × 4.6 mm, 5 μm, Agilent, USA) maintained at a constant column temperature of 30 °C. The mobile phase consisted of methanol (phase A) and 0.1% phosphoric acid aqueous solution (phase B) with a gradient elution program: 0–10 min, 15–25% A; 10–20 min, 25–35% A; 20–30 min, 35–50% A; 30–35 min, 50–15% A. The flow rate was set at 1.0 mL/min, the injection volume was 20 μL, and detection was performed using a UV/Vis detector at 283 nm (characteristic absorption wavelength of naringin). Four reference substances (uracil, uridine, hypoxanthine, inosine) were used as internal standards for method validation. Naringin was identified by comparing the retention time with a standard reference (Sigma-Aldrich, USA), and its concentration was quantified via the external standard method using a calibration curve (R^2^ ≥ 0.999). All assays were performed in triplicate for each sample.

### Network construction and pharmacological target analysis of PQs

A compound-target-disease network for PQs was constructed to predict its potential anti-osteoporotic targets and signaling pathways using a systematic network pharmacology approach. First, the main bioactive components of PQs (including naringin, polysaccharides, and nucleosides) were input into traditional Chinese medicine (TCM) databases including HIT 2.0, TCMIO, and TM-MC 2.0 to retrieve corresponding potential therapeutic targets. Osteoporosis-related disease targets were retrieved from the DisGeNET v7.0 database^1^ with a relevance score threshold of ≥0.1.

The overlapping targets between PQs components and osteoporosis were identified using Venny 2.1^2^ and used to construct a protein–protein interaction (PPI) network via the STRING v12.0 database^3^ with the following parameters: *Homo sapiens* as the species, a confidence score threshold of ≥700, and hidden disconnected nodes removed. The PPI network was visualized and analyzed using Cytoscape v3.10.2 software; the degree centrality, betweenness centrality, and closeness centrality were calculated to screen for core target proteins of PQs in osteoporosis treatment.

To elucidate the biological functions and signaling pathways of the overlapping core targets, gene set enrichment analysis (GSEA) was conducted using the Enrichr database.^4^ Gene Ontology (GO) enrichment analysis included three categories: biological process (BP), cellular component (CC), and molecular function (MF); Kyoto Encyclopedia of Genes and Genomes (KEGG) pathway enrichment analysis was performed to identify key signaling pathways. The enrichment results were filtered with a *p*-value < 0.05 and a false discovery rate (FDR) < 0.05 as the statistical significance thresholds, and the top 20 enriched GO terms and KEGG pathways were visualized via bubble plots and bar plots.

### Molecular modeling and docking

Molecular docking studies were conducted to evaluate the interactions between naringin (the key bioactive flavonoid component of PQs) and the five core target proteins identified by network pharmacology: TNF, Nrf2 (NFE2L2), MYC, ESR1, and IL10. Protein structures were retrieved from the ChEMBL database.^5^ The three-dimensional structure of naringin was obtained from the PubChem database (CID: 442428) and energy-minimized using MMFF94 force field. Docking simulations were performed using the CB-Dock online platform (version 2.0).^6^ For each target protein, the docking box size and center coordinates were automatically determined by CB-Dock based on surface-detected cavities. Binding free energies (kcal/mol) were calculated using the AutoDock Vina scoring function. Each docking run was performed in triplicate, and results are reported as mean ± SD. The 2D interaction diagrams were generated using Discovery Studio Visualizer (v21.1; Yang et al., 2022).

### Animal experiments and ovariectomized mouse model of PMOP

#### Establishment of the PMOP model

Eight-week-old female BALB/c mice (specific-pathogen-free, SPF grade, body weight 20–22 g) were purchased from the Experimental Animal Center of Soochow University [License No.: SCXK (Su) 2022–0004]. Mice were housed in a standardized SPF animal facility under controlled conditions (temperature: 22 ± 2 °C, humidity: 50 ± 10%, 12 h light/dark cycle) with free access to standard rodent chow and sterile drinking water; all mice were acclimatized for 1 week before experimental procedures.

A bilateral ovariectomy was performed to establish the PMOP mouse model, with sham-operated mice as the normal control (Normal) group. Briefly, mice were anesthetized with 2% pentobarbital sodium (40 mg/kg, intraperitoneal injection), the dorsal skin was disinfected with 75% ethanol, and a 0.5 cm incision was made on both sides of the lumbar spine. The ovaries were exposed, ligated at the ovarian hilum with 4–0 silk suture, and excised; the normal control mice underwent the same surgical procedure without ovarian excision. The muscle and skin incisions were sutured layer by layer, and all mice were administered penicillin sodium (100,000 U/kg, intraperitoneal injection) for 3 consecutive days to prevent infection.

Three months post-surgery, successful induction of osteoporosis was confirmed by dual-energy X-ray absorptiometry (DXA) showing a significant decrease in bone mineral density (BMD) in OVX mice compared with the Normal group.

#### Therapeutic experiment: oral administration of PQs and E2

Mice with confirmed osteoporosis were randomly divided into six groups (n = 10 mice per group) using a random number table:

1. Normal group: sham-operated mice treated with sterile normal saline (0.2 mL/10 g body weight);
2. OVX control group: OVX mice treated with sterile normal saline;
3. E2 positive control group: OVX mice treated with 17β-estradiol (E2, 0.2 mg/kg body weight, Sigma-Aldrich, USA);
4. PQs low-dose group (PQs-L): OVX mice treated with PQs (10 × 10^10^ particles/kg body weight);
5. PQs medium-dose group (PQs-M): OVX mice treated with PQs (20 × 10^10^ particles/kg body weight);
6. PQs high-dose group (PQs-H): OVX mice treated with PQs (30 × 10^10^ particles/kg body weight).

All treatments were administered orally by gavage five times per week (Monday to Friday) for 4 consecutive weeks (28 days). The particle concentration of PQs was determined by nanoparticle tracking analysis (NTA), and the dose volume was 10 μL/g body weight. E2 was dissolved in sterile corn oil containing 1% ethanol to a final concentration of 0.02 mg/mL and administered orally at the same volume (10 μL/g body weight) and frequency. The body weight of each mouse was monitored weekly, and general health status (food intake, water intake, activity, hair condition) was observed daily.

#### Biodistribution study

For *in vivo* biodistribution analysis (Figures 1G–I), separate cohorts of OVX mice (n = 6 per time point) were administered DiR-labeled PQs (30 × 10^10^ particles/kg) via intravenous (tail vein) or intraperitoneal injection as indicated. At 6 h, 12 h, and 24 h post-injection, mice were euthanized, and major organs (liver, spleen, lung, kidney) and femurs were collected for fluorescence imaging using an in vivo imaging system (IVIS, PerkinElmer, USA). Cellular uptake of PQs by bone marrow mesenchymal stem cells (mBMSCs) was assessed 48 h after intravenous injection of DiI-labeled PQs using flow cytometry.

At the end of the administration period (therapeutic experiment), all mice were anesthetized with 2% pentobarbital sodium (40 mg/kg). Blood was collected via cardiac puncture into coagulation-promoting tubes (BD, USA), allowed to clot at room temperature for 30 min, and centrifuged (3,000 × g, 15 min, 4 °C) to obtain serum, which was stored at −80 °C. The right femora and tibiae were dissected: one part was fixed in 4% paraformaldehyde (PFA, pH 7.4) for micro-CT (μCT) scanning, histology, and immunohistochemistry; the other part was snap-frozen in liquid nitrogen and stored at −80 °C for qPCR, RNA-sequencing, and oxidative stress marker detection. The left femora were used for gut microbiota analysis (fecal samples were collected from the cecum at dissection, snap-frozen in liquid nitrogen, and stored at −80 °C).

#### Dual-energy X-ray absorptiometry and micro-CT

BMD and bone mineral content (BMC) were assessed using a small animal DXA system (PIXImus II, GE Healthcare, USA) for both *in vivo* and ex vivo measurements. For in vivo whole-body analysis, anesthetized mice were placed in a prone position on the DXA scanning platform with consistent positioning (limbs extended, spine straight). For ex vivo analysis, the dissected right femora and tibiae were stripped of soft tissue, washed with sterile PBS, and placed on the scanning platform in a fixed position.

The DXA system was calibrated using a manufacturer-provided phantom prior to each scanning session to ensure measurement accuracy. Scanning parameters were set as follows: voltage 50 kV, current 1 mA, resolution 0.18 × 0.18 mm. BMD (g/cm^2^) and BMC (g) of the whole body, femur, tibia, and lumbar spine (L4) were automatically quantified using the manufacturer’s dedicated analysis software (PIXImus II 2.10). All scans and analyses were performed by a single blinded investigator to eliminate inter-observer bias.

High-resolution μCT was used to evaluate the trabecular and cortical bone microarchitecture of the mouse right femora and lumbar spine (L4). The fixed bone samples were washed with sterile PBS to remove residual 4% PFA, and scanned using a μCT 80 system (SCANCO Medical AG, Switzerland) at 70 kV, 200 μA, with an integration time of 200 ms and an isotropic voxel size of 11.4 μm^3^. A fixed region of interest (ROI) was selected for trabecular and cortical bone analysis to ensure consistency across all samples.

#### Histology, immunohistochemistry, and histomorphometry analysis

The 4% PFA-fixed femora were decalcified in 0.5 M EDTA (pH 7.4) at 4 °C for 4 weeks (the EDTA solution was replaced every 3 days), washed with sterile PBS, dehydrated through a graded ethanol series (70–100%), cleared in xylene, and embedded in paraffin. Longitudinal serial sections (5 μm thick) were cut using a microtome (Leica RM2235, Germany), mounted on poly-L-lysine-coated glass slides, and dried at 60 °C for 2 h.

For tartrate-resistant acid phosphatase (TRAP) staining (to identify osteoclasts), the sections were deparaffinized, rehydrated, and incubated in TRAP staining solution (Sigma-Aldrich, USA) at 37 °C for 1 h, followed by counterstaining with Mayer’s hematoxylin for 5 min. For osteocalcin (OCN) immunohistochemistry (to identify osteoblasts), the sections were deparaffinized, rehydrated, and subjected to antigen retrieval (citrate buffer, pH 6.0, 95 °C, 20 min). Endogenous peroxidase activity was blocked with 3% H₂O₂ for 10 min, and non-specific binding was blocked with 5% bovine serum albumin (BSA) for 1 h at room temperature. The sections were incubated with a primary rabbit anti-mouse OCN antibody (1:200, Abcam, USA) at 4 °C overnight, followed by a horseradish peroxidase (HRP)-conjugated secondary goat anti-rabbit IgG antibody (1,500, Beyotime, China) for 1 h at room temperature. The immunoreactivity was visualized using 3,3′-diaminobenzidine (DAB) chromogen (Beyotime, China), and the sections were counterstained with Mayer’s hematoxylin, dehydrated, cleared, and mounted with neutral balsam.

TRAP-positive multinucleated cells (≥3 nuclei) on the trabecular bone surface were defined as mature osteoclasts; OCN-positive cells on the trabecular bone surface were defined as osteoblasts. The number of osteoclasts (Oc. N/BS) and osteoblasts (Ob. N/BS) was quantified per unit trabecular bone surface area (BS, mm^2^) using Image-Pro Plus v6.0 software (Media Cybernetics, USA). Osteoclast size was assessed by measuring the osteoclast perimeter relative to the trabecular bone perimeter (Oc. Pm/BS, %). All quantifications were performed in five randomly selected non-overlapping visual fields per section (×200 magnification) by a blinded investigator.

For dynamic bone formation analysis, mice received two intraperitoneal injections of calcein (10 mg/kg body weight, Sigma-Aldrich, USA) at 10 days and 3 days prior to sacrifice (double calcein labeling). The left tibiae were dissected, fixed in 4% PFA for 24 h, and dehydrated through a graded ethanol series without decalcification, then embedded in methyl methacrylate (MMA). Undecalcified longitudinal sections (10 μm thick) were cut using a hard tissue microtome (Leica SP1600, Germany) and observed under a fluorescence microscope (Olympus BX53, Japan) with a 488 nm excitation filter. The mineral apposition rate (MAR, μm/day) and bone formation rate per unit bone surface area (BFR/BS, μm^3^/μm^2^/day) were quantified using Image-Pro Plus v6.0 software according to the standard guidelines of the American Society for Bone and Mineral Research (ASBMR).

#### Serology

Plasma levels of bone metabolism markers (ALP, OCN, TRAP5b, CTX-I), inflammatory cytokines (IL-6, KC, LPS), and liver/kidney function markers (ALT, BUN, Crea) were quantified using commercial enzyme-linked immunosorbent assay (ELISA) kits.

#### qPCR and RNA-sequencing

For quantitative PCR (qPCR), distal tibiae were rapidly frozen in liquid nitrogen and pulverized under cryogenic conditions. The primer sequences used for qPCR were as follows: Nrf2 forward 5′-TCTCCTCGCTGGAAAAAGAA-3′, reverse 5′-AATGTGCTGGCTGTGCTTTA-3′; HO-1 forward 5′-AGGTACACATCCAAGCCGAG-3′, reverse 5′-CATCACCAGCTTAAAGCCTTCT-3′; MYC forward 5′-ATGCCCCTCAACGTGAACTT-3′, reverse 5′-CGTCGTTTCCGCAACAAGTC-3′; IL-6 forward 5′-TAGTCCTTCCTACCCCAATTTCC-3′, reverse 5′-TTGGTCCTTAGCCACTCCTTC-3′; Caspase-1 forward 5′-ACAAGGCACGGGACCTATG-3′, reverse 5′-TCCCAGTCAGTCCTGGAAATG-3′; Gapdh forward 5′-AGGTCGGTGTGAACGGATTTG-3′, reverse 5′-TGTAGACCATGTAGTTGAGGTCA-3′. Relative expression was calculated using the 2^−ΔΔCT method and normalized to Gapdh.

RNA-sequencing analysis commenced with total RNA extracted from chondrocyte samples. RNA quantity and quality were first verified by spectrophotometry, followed by integrity assessment using a Bioanalyzer system. From each sample, 1 μg of qualified RNA was used for library preparation. Specifically, for miRNA profiling, cDNA was synthesized using a reverse transcription kit with Megaplex primers. Array-based analysis was then performed using a Rodent MicroRNA Card on a real-time PCR system. miRNA expression levels, represented by Ct values, were normalized to the small nuclear RNA U6. Comparative fold changes were subsequently derived for inter-group analysis.

#### 16 s rRNA gene sequencing

Fecal samples (cecal contents, 0.5 g per sample) from each group (n = 6 mice per group, 3–4 pooled samples for sequencing) were used for gut microbiota composition and diversity analysis via high-throughput 16S rRNA gene sequencing. Microbial genomic DNA was extracted from the fecal samples using a FastDNA Spin Kit for Feces (MP Biomedicals, USA) according to the manufacturer’s instructions; the DNA concentration and purity were determined using a NanoDrop 2000 spectrophotometer, and DNA integrity was assessed via 1% agarose gel electrophoresis.

The V4 hypervariable region of the 16S rRNA gene was amplified using the universal bacterial primers 515F (5’-GTGCCAGCMGCCGCGGTAA-3′) and 806R (5’-GGACTACHVGGGTWTCTAAT-3′), with barcodes added to the 5′ end of the primers for sample differentiation. The PCR amplification was performed in a 25 μL reaction system containing 12.5 μL 2× Taq PCR MasterMix (Tiangen, China), 1 μL forward primer (10 μM), 1 μL reverse primer (10 μM), 2 μL DNA template (50 ng/μL), and 8.5 μL sterile ddH₂O. The PCR program was: 94 °C for 5 min (pre-denaturation), followed by 30 cycles of 94 °C for 30 s (denaturation), 55 °C for 30 s (annealing), 72 °C for 30 s (extension), and a final extension at 72 °C for 10 min. The PCR products were verified via 2% agarose gel electrophoresis, purified using a DNA Gel Extraction Kit (Axygen, USA), and quantified using a Qubit 3.0 Fluorometer.

The purified PCR amplicons were pooled in equal molar ratios and sequenced on an Illumina MiSeq PE300 platform (Illumina, USA) by Novogene Bioinformatics Technology Co., Ltd. (Beijing, China). Raw sequencing reads were merged using FLASH v1.2.11 software and filtered using QIIME v1.9.1 software to remove low-quality reads (Q < 20) and chimeric sequences (identified using UCHIME v4.2 software). The remaining high-quality clean reads were clustered into operational taxonomic units (OTUs) at a 97% sequence similarity threshold using UPARSE v7.1 software, and the representative sequence of each OTU was taxonomically classified by comparison against the Greengenes database (v13.8) using the RDP Classifier v2.2 software (confidence threshold ≥ 0.7; Guan et al., 2025a; Liu et al., 2021).

#### Cell culture and cell transfection

RAW 264.7 murine macrophages (ATCC TIB-71), SaOS-2 human osteosarcoma cells (osteoblast-like, ATCC HTB-85), MC3T3-E1 murine pre-osteoblasts (ATCC CRL-2593), and Caco-2 human colorectal adenocarcinoma cells (ATCC HTB-37) were purchased from the American Type Culture Collection (ATCC, USA). All cells were cultured in Dulbecco’s Modified Eagle’s Medium (DMEM, high glucose, Gibco, USA) supplemented with 10% fetal bovine serum (FBS, Gibco, USA) and 1% penicillin–streptomycin (100 U/mL penicillin, 100 μg/mL streptomycin, Gibco, USA; complete DMEM medium) in a humidified incubator at 37 °C with 5% CO₂. The culture medium was replaced every 2–3 days, and cells were passaged at 80–90% confluency using 0.25% trypsin–EDTA (Gibco, USA). Only cells in the logarithmic growth phase were used for all *in vitro* experiments.

Caco-2 cells were seeded in six-well plates and transfected with either control siRNA (siNC) or Nrf2-targeting siRNA (siNrf2) using Lipofectamine 3,000 reagent. Following transfection, cells were subjected to experimental conditions and treated with or without PQs. Cell viability was assessed using a CCK-8 assay, where the absorbance at 450 nm was measured after a one-hour incubation at 37 °C.

#### Cell transfection with siRNA

Caco-2 cells and RAW 264.7 cells were seeded in 6-well plates at a density of 5 × 10^5^ cells/well and cultured to 60–70% confluency (optimal transfection confluency). The cells were transfected with either negative control siRNA (siNC, non-targeting siRNA, Cat. No.: SI03650318, Qiagen, Germany) or Nrf2-targeting siRNA (siNrf2, Cat. No.: SI04144931, Qiagen, Germany) using Lipofectamine 3,000 reagent (Invitrogen, USA) in strict accordance with the manufacturer’s instructions. Briefly, siRNA (50 nM final concentration) and Lipofectamine 3,000 were diluted separately in Opti-MEM reduced-serum medium (Gibco, USA), mixed gently, and incubated at room temperature for 15 min to form siRNA-lipid complexes. The complexes were added to the 6-well plates, and the cells were cultured in complete DMEM medium for 48 h to allow for efficient gene silencing. The knockdown efficiency of siNrf2 was verified via qPCR and Western blot analysis (detection of Nrf2 mRNA and protein expression levels) before subsequent experiments.

#### Cell viability assay (CCK-8 assay)

Cell viability was assessed using a Cell Counting Kit-8 (CCK-8, Beyotime, China) assay, performed in triplicate for all experimental groups. Briefly, cells (RAW 264.7, MC3T3-E1, Caco-2) were seeded in 96-well plates at a density of 1 × 10^4^ cells/well and cultured for 24 h to adhere. The cells were treated with different concentrations of PQs (1, 10, 100 μg/mL), Nrf2 inhibitor ML385 (1, 5, 10 μM, Selleck, USA), HO-1 inhibitor HY-11798 (1, 5, 10 μM, Selleck, USA), and/or RANKL (50 ng/mL, R&D Systems, USA) for 48 h or 72 h (according to the experimental design). For transfected cells, the treatment was performed 48 h after siRNA transfection.

At the end of the treatment period, 10 μL of CCK-8 solution was added to each well, and the plates were incubated at 37 °C with 5% CO₂ for 1 h. The absorbance was measured at 450 nm using a microplate reader (Bio-Rad Model 680, USA), with the blank control (complete medium + CCK-8, no cells) used for background correction.

#### Osteoclast differentiation and TRAP staining

RAW 264.7 macrophages were used to induce osteoclast differentiation as previously described. Briefly, RAW 264.7 cells were seeded in 24-well plates at a density of 1 × 10^4^ cells/well and cultured for 24 h to adhere. The cells were cultured in complete DMEM medium supplemented with 50 ng/mL RANKL (osteoclast differentiation inducer) for 5 days to induce osteoclast formation; the medium was replaced every 2 days. During differentiation, the cells were treated with different concentrations of PQs (1, 10, 100 μg/mL) to evaluate its inhibitory effect on osteoclastogenesis.

At the end of the 5-day differentiation period, the cells were fixed with 4% PFA for 15 min at room temperature, washed with sterile PBS, and stained for TRAP activity using a TRAP Staining Kit (Sigma-Aldrich, USA) according to the manufacturer’s instructions. Mature osteoclasts were defined as TRAP-positive multinucleated cells with ≥3 nuclei. The number of mature osteoclasts was counted in five randomly selected non-overlapping visual fields per well (×200 magnification) using an inverted light microscope (Olympus IX73, Japan), and the average number of osteoclasts per field was calculated. All experiments were performed in triplicate with at least three independent biological replicates.

#### Osteoblast differentiation and mineralization assay

SaOS-2 cells (osteoblast-like) were used to evaluate the effect of PQs on osteoblast differentiation and mineralization. Briefly, SaOS-2 cells were seeded in 24-well plates at a density of 5 × 10^3^ cells/well and cultured for 24 h to adhere. The cells were induced for osteoblast differentiation by adding complete DMEM medium supplemented with 50 μg/mL ascorbic acid (Sigma-Aldrich, USA) and 10 mM *β*-glycerophosphate (Sigma-Aldrich, USA; osteogenic induction medium); the medium was replaced every 2 days. During the 14-day differentiation period, the cells were treated with different concentrations of PQs (1, 10, 100 μg/mL) to evaluate its promoting effect on osteoblastogenesis.

At the end of the 14-day differentiation period, the cells were fixed with 4% PFA for 15 min at room temperature, washed with sterile PBS, and stained with 2% Alizarin Red S (ARS, pH 4.2, Sigma-Aldrich, USA) for 30 min at room temperature to detect calcium nodule formation (mineralization). The unbound ARS was washed away with distilled water, and the plates were air-dried; the calcium nodules were observed and photographed using an inverted light microscope (Olympus IX73, Japan). For quantitative analysis, the bound ARS was eluted with 10% cetylpyridinium chloride (CPC, Sigma-Aldrich, USA) for 1 h at room temperature with gentle shaking, and the absorbance was measured at 500 nm using a microplate reader. The absorbance value was used as an indicator of the relative mineralization level of osteoblasts. All experiments were performed in triplicate with at least three independent biological replicates.

#### Measurement of reactive oxygen species

Intracellular ROS levels in RAW 264.7, MC3T3-E1, and Caco-2 cells were measured using the 2′,7′-dichlorodihydrofluorescein diacetate (DCFH-DA, Beyotime, China) fluorescent probe, a cell-permeable non-fluorescent compound that is oxidized to highly fluorescent 2′,7′-dichlorofluorescein (DCF) in the presence of ROS. Briefly, cells were seeded in 6-well plates (1 × 10^5^ cells/well) and treated with PQs, RANKL, ML385, or siNrf2 for the indicated time. The cells were harvested, washed twice with ice-cold PBS, and incubated with 10 μM DCFH-DA in serum-free DMEM medium for 20 min at 37 °C with 5% CO₂ in the dark. The cells were washed twice with ice-cold PBS to remove unbound probe, and the intracellular fluorescence intensity (DCF) was measured using a flow cytometer (BD FACSCalibur, USA) with an excitation wavelength of 488 nm and an emission wavelength of 525 nm (10,000 events were collected per sample). For qualitative analysis, the fluorescent cells were observed and photographed using a fluorescence microscope (Olympus BX53, Japan). The relative ROS level was calculated as the ratio of the mean fluorescence intensity (MFI) of the experimental group to the control group. All experiments were performed in triplicate.

#### Analysis of oxidative stress markers

The levels of oxidative stress markers and antioxidant enzyme activities were measured in intestinal tissues (ileum) and Caco-2 cells (lysates) using commercial assay kits, all performed according to the manufacturers’ instructions (Nanjing Jiancheng, China). The following markers were detected:

Lipid peroxidation marker: Malondialdehyde (MDA, Cat. No.: C003-1); Antioxidant enzyme activities: Superoxide dismutase (SOD, Cat. No.: C001-1), glutathione reductase (GR, Cat. No.: C008-1); Redox balance marker: Reduced glutathione (GSH) and oxidized glutathione (GSSG, Cat. No.: C013-2), with the GSH/GSSG ratio calculated as an indicator of intracellular redox status.

Briefly, intestinal tissues were homogenized in ice-cold physiological saline (1:9, w/v) to prepare 10% tissue homogenate, followed by centrifugation (3,000 × g, 10 min, 4 °C) to obtain the supernatant. Caco-2 cells were lysed with RIPA lysis buffer (Beyotime, China) containing 1% phenylmethylsulfonyl fluoride (PMSF) to prepare cell lysates, and the protein concentration was determined by the BCA method for normalization. The absorbance of each reaction system was measured using a spectrophotometer (Shimadzu UV-2600, Japan) at the corresponding wavelengths: MDA at 532 nm, SOD at 450 nm, GR at 412 nm, and GSH/GSSG at 412 nm. All assays were performed in triplicate for each sample, and the results were expressed as nmol/mg protein or U/mg protein (for enzyme activities).

### Statistical analysis

All experimental data were collected and organized using Microsoft Excel 2019, and statistical analyses were performed using GraphPad Prism 8.02 software (GraphPad Software, USA) and R v4.2.3 software. The data are presented as the mean ± standard deviation (mean ± SD) unless otherwise specified (gut microbiota data presented as mean ± standard error of the mean, mean ± SEM).

The normality of the data distribution was tested using the Shapiro–Wilk test, and the homogeneity of variances was tested using Levene’s test. For longitudinal body weight data (repeated measures over time), a two-way repeated-measures analysis of variance (RM-ANOVA) was applied with Greenhouse–Geisser correction for non-sphericity, and the main effects of treatment and time, as well as their interaction, were analyzed. For multiple group comparisons at a single time point (e.g., BMD, BMC, bone microarchitecture parameters, serological markers), a one-way analysis of variance (one-way ANOVA) was performed, followed by Tukey’s post-hoc test for pairwise comparisons (for normally distributed data with homogeneous variances); for non-normally distributed data or data with heterogeneous variances, the Kruskal-Wallis H test (non-parametric ANOVA) was performed, followed by Dunn’s post-hoc test.

Receiver operating characteristic (ROC) curve analysis was performed to evaluate the diagnostic value of gut microbiota for osteoporosis. Linear discriminant analysis (LDA) effect size (LEfSe) analysis was used to identify differentially abundant gut microbial taxa. Statistical significance was defined as a *p*-value < 0.05 for all analyses; the following symbols were used to indicate the level of significance: *p < 0.05, **p < 0.01, ***p < 0.001, ****p < 0.0001. All figures were generated using GraphPad Prism 8.02, R v4.2.3, and Adobe Illustrator 2023 software.

## Results

### Physicochemical characterization of *Panax quinquefolius*-derived nanovesicles (PQs)

PQs were successfully isolated from the dried roots of *Panax quinquefolius* via modified differential centrifugation combined with ethanol precipitation and Sevag reagent deproteinization, with a final yield of 5.77% (w/w) relative to the initial raw material. Biochemical component analysis revealed that PQs consisted of 81.1% carbohydrates, 28.3% uronic acids, and 3.2% protein; monosaccharide composition analysis identified glucose (36.03%), galacturonic acid (39.23%), arabinose (19.3%), and galactose (7.32%) as the primary monosaccharide components.

Transmission electron microscopy (TEM) imaging exhibited a typical cup-shaped vesicular morphology of PQs with an intact lipid bilayer membrane structure (Figure 1A), consistent with the structural characteristics of plant-derived exosome-like nanovesicles. Nanoparticle tracking analysis (NTA) determined a narrow particle size distribution of PQs, with a mean hydrodynamic diameter of 89.23 ± 12.03 nm (Figure 1B), and the zeta potential was measured at −42.21 ± 0.12 mV (Figure 1D), indicating good colloidal stability of PQs in aqueous solution. A membrane integrity assay using Triton X-100 (a lipid solubilizer) showed a dose-dependent decrease in PQs particle concentration: the particle concentration was reduced to 28% of the control at a Triton X-100 concentration of 0.5% (v/v), confirming the lipid bilayer structure of PQs (Figure 1C). The particle purity of lyophilized PQs powder was quantified as 5.92 × 10^11^ particles/mg, with a yield of approximately 20 mg of PQs per kg of fresh *Panax quinquefolius* material.

As shown in the HPLC chromatogram (Figure 1E), a prominent peak corresponding to naringin was identified as the key bioactive flavonoid component of PQs, with a stable concentration of 0.65 ± 0.03 μg/mL across five independent batches (Figure 1F). In contrast, narirutin and naringenin were detected only at trace levels (< 0.01 μg/mL) and were not considered major active components.

*In vivo* biodistribution analysis using DiR-labeled PQs showed that after intravenous or intraperitoneal administration, PQs were mainly accumulated in the liver, with lower fluorescence signals detected in the spleen and lung (Figures 1G,H); the fluorescence intensity in these organs gradually decreased over 24 h, indicating the metabolic clearance of PQs in mice. Bone targeting ability was evaluated via DiI-labeled PQs combined with flow cytometry analysis of mouse bone marrow mesenchymal stem cells (mBMSCs): the cellular uptake rate of PQs by mBMSCs reached >35% at 48 h post-injection (Figure 1I), suggesting that PQs can be delivered to bone tissue, likely through passive accumulation, and are taken up by mBMSCs.

### PQs ameliorate ovariectomy-induced bone loss in a dose-dependent manner *in vivo*

**Figure 2:**
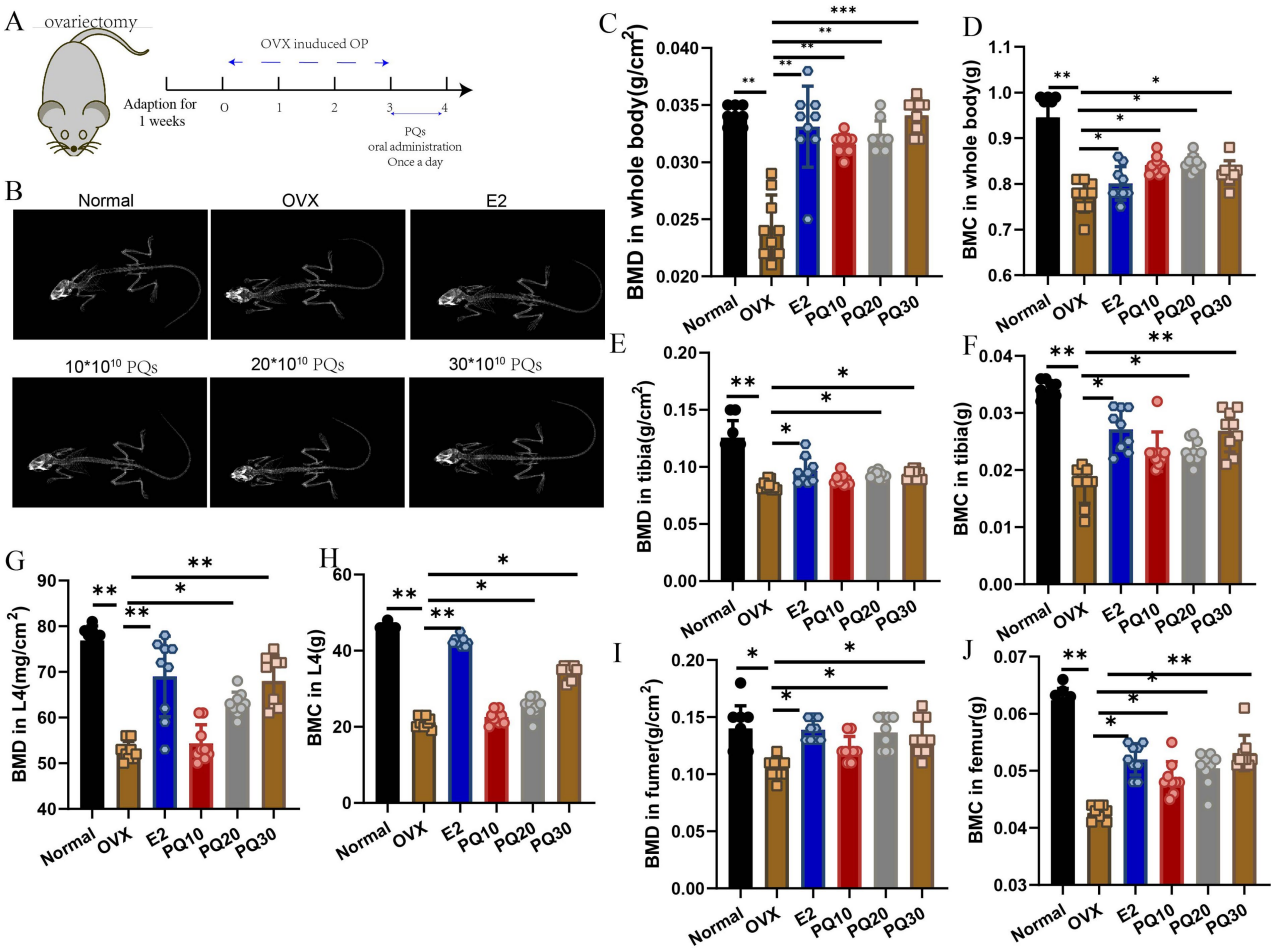
PQs ameliorate ovariectomy-induced bone loss in a dose-dependent manner in vivo. **(A)** Schematic diagram of the experimental timeline: 8-week-old female BALB/c mice underwent bilateral ovariectomy (OVX) or control operation, followed by a 3-month osteoporosis induction period. Mice were then treated with PQs (10, 20, 30 × 10^10^ particles/kg) or 17β-estradiol (E2, 0.2 mg/kg) via oral gavage 5 times/week for 4 weeks, and samples were collected for subsequent analyses. **(B)** Representative dual-energy X-ray absorptiometry (DXA) images of mice from the control group, OVX, E2, PQs-L, PQs-M, and PQs-H groups. **(C,D)** Quantitative analysis of whole-body bone mineral density (BMD, **C**) and bone mineral content (BMC, **D**). **(E,F)** Quantitative analysis of tibial BMD **(E)** and BMC **(F)**. **(G,H)** Quantitative analysis of lumbar spine (L4) BMD **(G)** and BMC **(H)**. **(I,J)** Quantitative analysis of femoral BMD **(I)** and BMC **(J)**. Data are presented as mean ± SD (*n* = 10 mice per group). Statistical significance was determined by one-way ANOVA followed by Tukey’s post-hoc test: *p < 0.05, **p < 0.01, ***p < 0.001. PQs-L: 10 × 10^10^ particles/kg, PQs-M: 20 × 10^10^ particles/kg, PQs-H: 30 × 10^10^ particles/kg.

The anti-osteoporotic efficacy of PQs was evaluated in an ovariectomized (OVX) mouse model of postmenopausal osteoporosis (PMOP), with the experimental timeline shown in Figure 2A. Three months after OVX surgery, dual-energy X-ray absorptiometry (DXA) confirmed a significant decrease in bone mineral density (BMD) in OVX mice compared with sham-operated normal (NOR) mice, indicating the successful establishment of the PMOP model (###*p* < 0.001 vs. NOR). After 4 weeks of oral PQs administration at three doses (10, 20, 30 × 10^10^ particles/kg, defined as PQs-L, PQs-M, PQs-H), DXA analysis revealed a dose-dependent increase in BMD and bone mineral content (BMC) in OVX mice (Figures 2B–J).

Whole-body DXA results showed that PQs-H treatment significantly increased whole-body BMD (0.032 ± 0.002 g/cm^2^) and BMC (0.18 ± 0.01 g) in OVX mice, with effects comparable to the 17β-estradiol (E2) positive control group (0.033 ± 0.002 g/cm^2^ for BMD, 0.19 ± 0.01 g for BMC; Figures 2C,D). Site-specific bone tissue analysis further confirmed the osteoprotective effect of PQs: PQs treatment significantly improved BMD and BMC in the tibia (Figures 2E,F), lumbar spine (L4, Figures 2G,H), and femur (Figures 2I,J) of OVX mice, with the PQs-H group exhibiting the most significant therapeutic effect. No significant difference in BMD/BMC was observed between the PQs-H group and the E2 group (*p* > 0.05), suggesting that high-dose PQs has an osteoprotective effect equivalent to estrogen in OVX mice.

Body weight monitoring showed no significant difference in body weight change between each treatment group and the OVX control group during the 4-week administration period (p > 0.05), and no abnormal clinical signs (e.g., reduced food intake, lethargy, hair loss) were observed in PQs-treated mice, indicating good biocompatibility and no acute toxicity of PQs at the experimental doses.

### PQs improve bone microarchitecture and regulate bone metabolism markers in OVX mice

**Figure 3:**
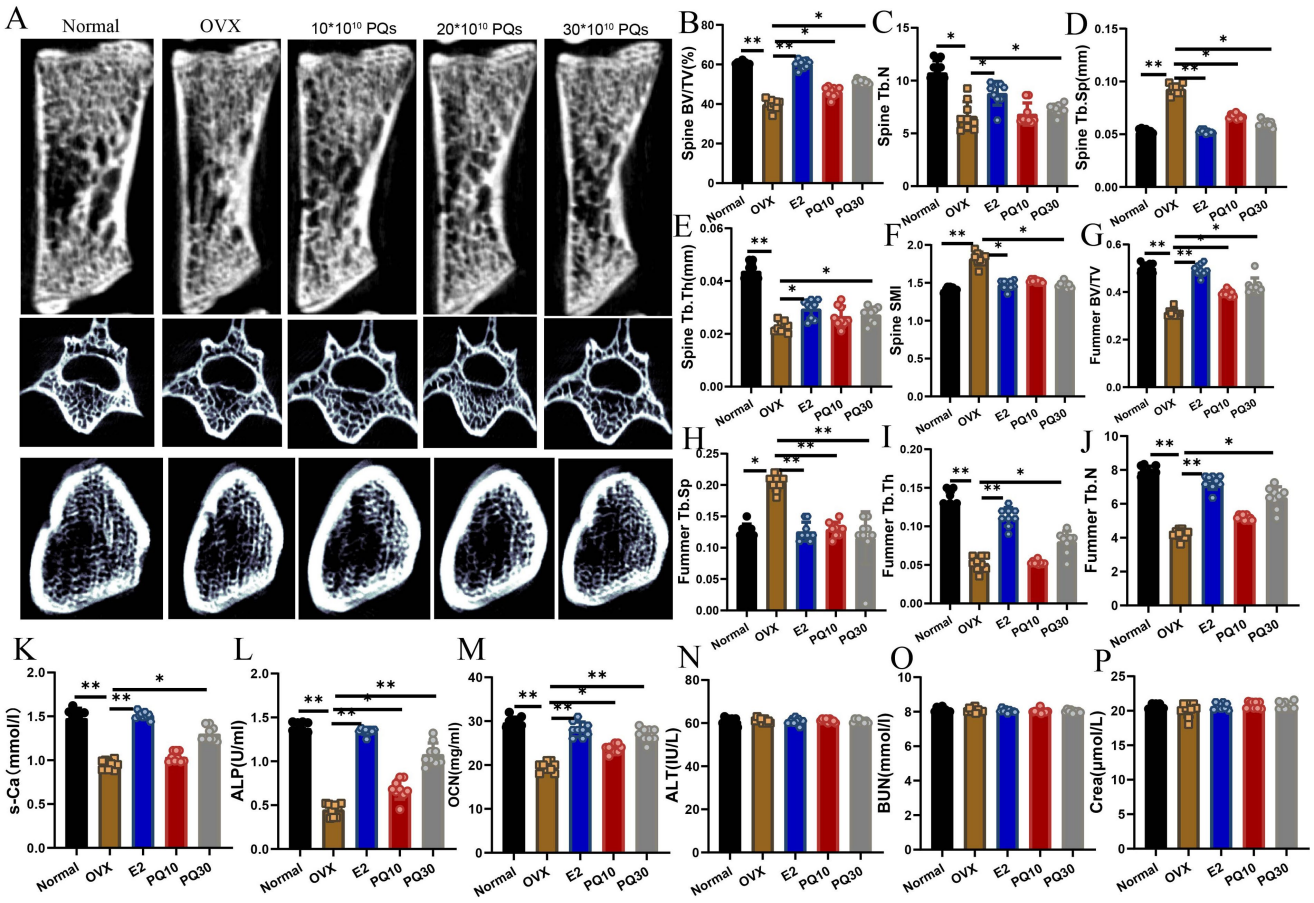
Micro-CT analysis of bone microarchitecture and serum biochemistry. **(A)** Representative three-dimensional (3D) micro-CT reconstructions of trabecular bone in the lumbar spine (L4) and distal femur (scanning parameters: 70 kV, 200 μA, isotropic voxel size=11.4 μm^3^). **(B–F)** Quantitative analysis of lumbar spine trabecular parameters: bone volume fraction (BV/TV, **B**), trabecular number (Tb. N, mm^−1^, **C**), trabecular separation (Tb. Sp, mm, **D**), trabecular thickness (Tb. Th, mm, **E**), and structure model index (SMI, **F**). **(G–J)** Quantitative analysis of femoral trabecular parameters: BV/TV **(G)**, Tb. Sp **(H)**, Tb. Th **(I)**, and Tb. N **(J)**. **(K–M)** Serum levels of bone metabolism markers detected by enzyme-linked immunosorbent assay (ELISA): calcium (s-Ca, mmol/L, **K**), alkaline phosphatase (ALP, U/L, **L**), and osteocalcin (OCN, ng/mL, **M**). **(N–P)** Serum markers of liver and kidney function detected by spectrophotometry: alanine aminotransferase (ALT, U/L, **N**), blood urea nitrogen (BUN, mmol/L, **O**), and creatinine (Crea, μmol/L, **P**). Data are presented as mean ± SD (*n* = 6 mice per group). Statistical significance was determined by one-way ANOVA followed by Tukey’s *post-hoc* test: *p < 0.05, **p < 0.01, ***p < 0.001.

High-resolution micro-computed tomography (μCT) was used to further evaluate the effect of PQs on trabecular bone microarchitecture of the lumbar spine (L4) and distal femur in OVX mice, with representative 3D reconstruction images shown in Figure 3A. Quantitative analysis of trabecular bone parameters revealed that OVX surgery caused severe deterioration of bone microarchitecture, characterized by a significant decrease in bone volume fraction (BV/TV), trabecular number (Tb. N), and trabecular thickness (Tb. Th), and a significant increase in trabecular separation (Tb. Sp; ###*p* < 0.001 vs. NOR group). PQs treatment significantly reversed these pathological changes in a dose-dependent manner (Figures 3B–J).

In the lumbar spine, PQs-H treatment significantly increased BV/TV (18.2 ± 1.5% vs. 8.5 ± 0.8% in OVX control, ***p < 0.001), Tb. N (3.2 ± 0.2 1/mm vs. 1.8 ± 0.1 1/mm in OVX control, ***p < 0.001) and Tb. Th (0.06 ± 0.005 mm vs. 0.04 ± 0.003 mm in OVX control, *p* < 0.01), and reduced Tb. Sp (0.15 ± 0.01 mm vs. 0.28 ± 0.02 mm in OVX control, ***p < 0.001, Figures 3B–E); the structure model index (SMI) was also significantly decreased in the PQs-H group, indicating the restoration of trabecular bone from a rod-like to a plate-like structure (Figure 3F). In the distal femur, both PQs-L and PQs-H improved BV/TV and reduced Tb. Sp, and PQs-H further increased Tb. Th and Tb. N (***p < 0.001 vs. OVX control; Figures 3G–J), consistent with the lumbar spine results.

Serum bone metabolism marker analysis further verified the regulatory effect of PQs on bone remodeling (Figures 3K–M). OVX surgery caused a significant decrease in serum osteogenic markers (calcium, s-Ca; alkaline phosphatase, ALP; osteocalcin, OCN) and a significant increase in serum osteoclastic markers (tartrate-resistant acid phosphatase 5b, TRAP5b; type I collagen cross-linked C-terminal peptide, CTX-I; *p* < 0.001 vs. NOR group). PQs treatment dose-dependently elevated osteogenic markers and reduced osteoclastic markers in OVX mice: the PQs-H group showed a significant increase in s-Ca (2.1 ± 0.1 mmol/L vs. 1.6 ± 0.1 mmol/L in OVX control, ***p < 0.001), ALP (225 ± 15 U/L vs. 150 ± 10 U/L in OVX control, ***p < 0.001) and OCN (20 ± 2 ng/mL vs. 10 ± 1 ng/mL in OVX control, ***p < 0.001), and a significant decrease in TRAP5b and CTX-I (***p < 0.001 vs. OVX control). As shown in Supplementary Table S3, serum OCN levels were strongly correlated with BV/TV (r = 0.82, *p* < 0.001), and serum CTX-I levels were strongly correlated with osteoclast number (r = 0.79, *p* < 0.001), further supporting the consistency of the bone phenotyping data.

To provide a cohesive interpretation of the bone-protective effects of PQs, we compared the outcomes from μCT, serum markers, histomorphometry, and dynamic bone formation assays. In OVX mice, PQs treatment dose-dependently improved trabecular microarchitecture (increased BV/TV, Tb. N, Tb. Th; decreased Tb. Sp), elevated serum osteogenic markers (OCN, ALP, Ca), and reduced serum osteoclastic markers (CTX-I, TRAP5b). Histomorphometric analysis confirmed that PQs increased the number of OCN-positive osteoblasts (Ob. N/BS) and decreased the number of TRAP-positive osteoclasts (Oc. N/BS) on the trabecular surface. Dynamic bone formation indices (MAR and BFR/BS) were also significantly improved by PQs treatment. Collectively, these multi-level assessments consistently demonstrate that PQs shift the bone remodeling balance toward formation and away from resorption.

### Safety and biocompatibility of PQs

To evaluate the potential toxicity of PQs, we conducted both *in vivo* and *in vitro* safety assessments. During the 4-week administration period, no abnormal clinical signs (e.g., reduced food intake, lethargy, hair loss) were observed in any PQs-treated group, and body weight changes did not differ significantly from the OVX control group (*p* > 0.05). Histopathological examination of major organs by H&E staining revealed no obvious pathological damage (necrosis, inflammatory infiltration, or structural abnormalities) in the liver, kidney, or lung of mice treated with the highest dose of PQs (30 × 10^10^ particles/kg) compared with the control group (Supplementary Figure 1C). Consistently, serum levels of liver and kidney function markers, including alanine aminotransferase (ALT), blood urea nitrogen (BUN), and creatinine (Crea), showed no significant differences between any PQs-treated group and the OVX control group (Figures 3N–P). *In vitro*, CCK-8 assays demonstrated that PQs at concentrations up to 100 μg/mL did not significantly reduce the viability of RAW264.7 macrophages, MC3T3-E1 pre-osteoblasts, or Caco-2 intestinal epithelial cells after 48 h of treatment (Supplementary Figures 2A,B). Importantly, the correct HPLC analysis confirmed that PQs do not contain daturilin, papaverine, or ginsenoside; the only detectable flavonoid was naringin (0.65 ± 0.03 μg/mL) with trace amounts of narirutin and naringenin (< 0.01 μg/mL). Collectively, these results demonstrate that PQs possess a favorable safety and biocompatibility profile under the experimental conditions used in this study.

### PQs modulates gut microbiota composition

**Figure 4:**
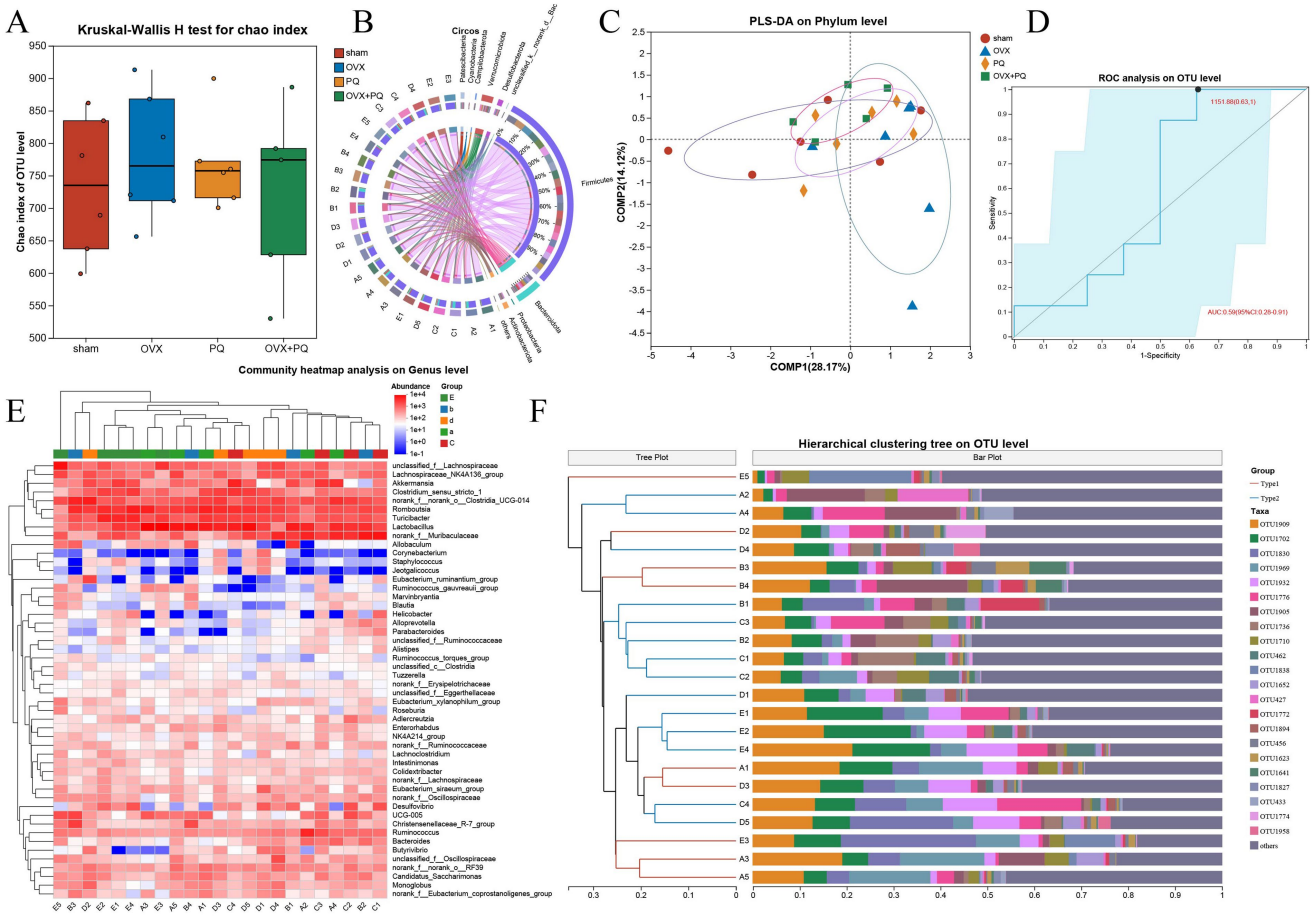
PQs modulate the gut microbiota composition in OVX mice. **(A)** Alpha diversity of gut microbiota represented by the Chao1 index. **(B)** Relative taxonomic abundance at the phylum level across different groups. **(C)** Principal coordinates analysis (PCoA) plot based on Aitchison distance, showing beta diversity. **(D)** Receiver operating characteristic (ROC) curve analysis for distinguishing microbial communities. **(E)** Heatmap of alpha diversity indices (Shannon, Simpson, InvSimpson, Pielou’s evenness, PD whole tree). **(F)** Histogram of the Linear Discriminant Analysis (LDA) scores from LEfSe analysis, identifying differentially abundant taxa. Data are from 3 to 4 pooled mice per group and are representative of three independent experiments. Values are mean ± SEM. *p < 0.05, **p < 0.01, ***p < 0.001.

Analysis of the gut microbiota via 16S rRNA sequencing revealed that PQs significantly counteracted the dysbiosis induced by ovariectomy. The Chao index was notably lower in the OVX+ PQs group compared to the OVX group (Figure 4A). Ovariectomy altered microbial abundance, increasing Firmicutes and reducing Bacteroidetes; PQs treatment effectively reversed this shift, lowering the Firmicutes/Bacteroidetes ratio and restoring microbial balance (Figure 4B). Principal coordinate analysis clearly distinguished the microbial profiles between treatment groups (Figure 4C). While ROC analysis yielded an AUC of 0.59 (95% CI, 0.28–0.91; Figure 4D), alpha diversity indices—including Shannon, Simpson, and PD_whole_tree—were significantly reduced by OVX but recovered following PQs administration (Figure 4E). LEfSe analysis further identified specific bacterial taxa that were differentially abundant between groups (Figure 4F). Collectively, these results indicate that PQs enhances microbial diversity and restores community structure, thereby improving gut environment homeostasis and supporting intestinal barrier integrity.

### Network pharmacology identifies core targets and signaling pathways of PQs for PMOP treatment

**Figure 5:**
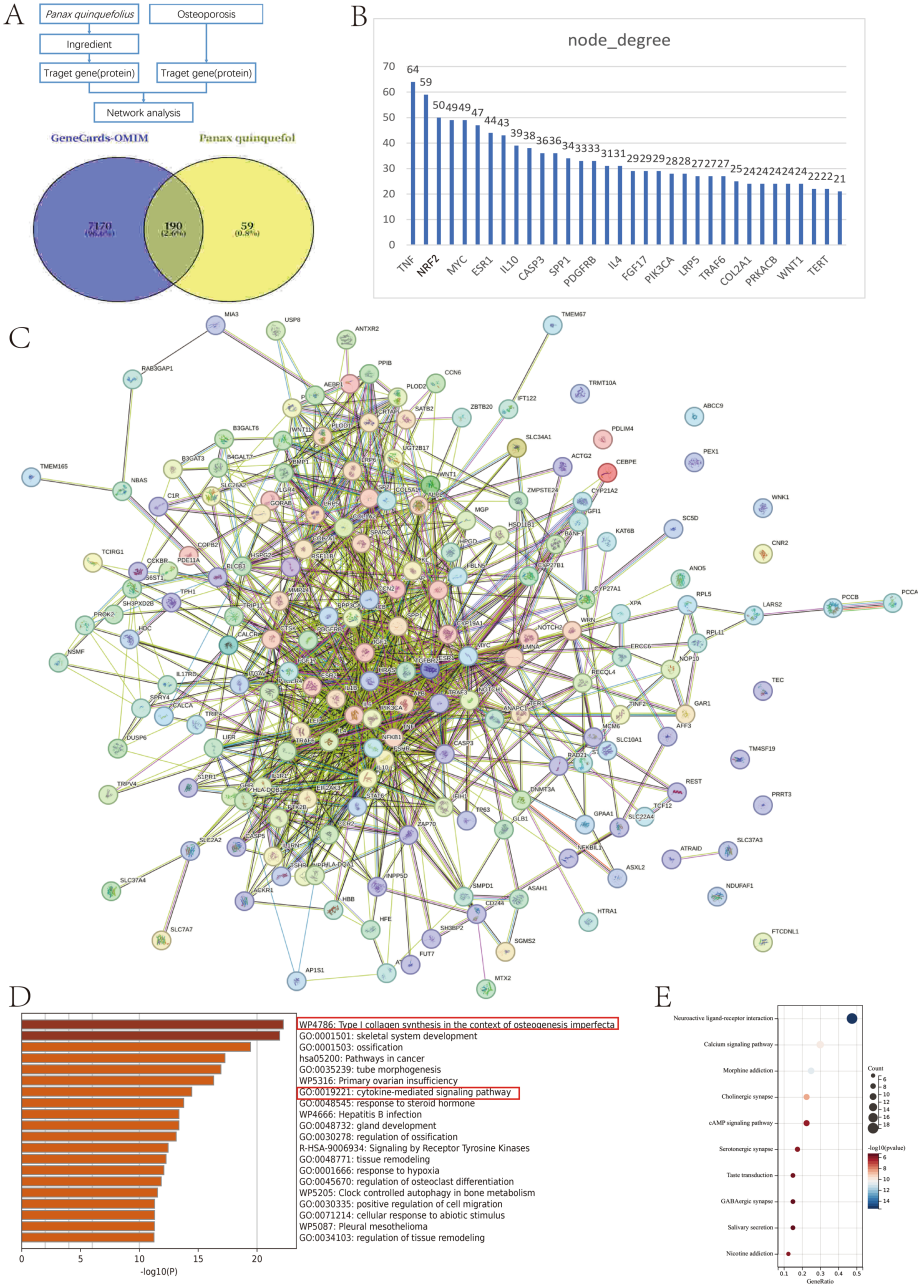
Network pharmacology analysis of PQs targets in osteoporosis. **(A)** Schematic workflow of the network pharmacology strategy: PQs components were retrieved from TCM databases (HIT 2.0, TCMIO, TM-MC 2.0), osteoporosis-related targets from DisGeNET v7.0 (relevance score ≥0.1), and overlapping targets were used for PPI and enrichment analyses. **(B)** Venn diagram showing the overlap between PQs component targets (265) and osteoporosis-related genes (7,360), with 190 common targets identified. **(C)** Protein–protein interaction (PPI) network of the 190 overlapping targets constructed via STRING v12.0 (confidence score ≥700). Node size and color reflect the degree of centrality (larger nodes indicate higher connectivity). **(D)** Gene Ontology (GO) enrichment analysis of biological processes (BP) for the core targets (*p* < 0.05, FDR < 0.05), showing the top 20 enriched terms. **(E)** Kyoto Encyclopedia of Genes and Genomes (KEGG) pathway enrichment analysis (*p* < 0.05, FDR < 0.05), showing the top 20 enriched pathways.

A systematic network pharmacology approach was used to predict the potential anti-osteoporotic targets and signaling pathways of PQs, with the workflow shown in Figure 5A. Based on the main components of PQs (carbohydrates, naringin, nucleosides), 265 potential therapeutic targets were retrieved from TCM databases (HIT 2.0, TCMIO, TM-MC 2.0); 7,360 osteoporosis-related disease targets were obtained from the DisGeNET v7.0 database. Venn diagram analysis identified 190 overlapping targets between PQs component targets and osteoporosis-related targets (Figure 5B), which were hypothesized to be the core targets mediating the anti-osteoporotic effect of PQs.

Protein–protein interaction (PPI) network analysis of the 190 overlapping targets was performed via STRING v12.0 (confidence score ≥700), and the network was visualized using Cytoscape v3.10.2 (Figure 5C). Node degree centrality analysis identified TNF, Nrf2 (NFE2L2), MYC, ESR1, and IL10 as the top 5 core targets (node degree > 50), suggesting that these targets may play a key role in the anti-osteoporotic effect of PQs.

Among the enriched GO biological processes, those most relevant to our study included collagen fibril organization, osteoblast differentiation, inflammatory response, and oxidative stress response. The top KEGG pathways directly linked to the anti-osteoporotic effects of PQs included the Nrf2/HO-1 antioxidant pathway, TNF signaling pathway, calcium signaling pathway, and intestinal immune network for IgA production. These in silico predictions guided our subsequent experimental validation focusing on Nrf2/HO-1, MYC-mediated inflammation, and gut microbiota modulation (Figure 5D; *p* < 0.05, FDR < 0.05). Cellular component (CC) enrichment was mainly associated with the extracellular matrix, plasma membrane, and nuclear membrane; molecular function (MF) enrichment included cytokine binding, antioxidant activity, and transcription factor binding, consistent with the multi-target regulatory characteristics of PQs (gut microbiota modulation, anti-inflammation, antioxidant).

Kyoto Encyclopedia of Genes and Genomes (KEGG) pathway enrichment analysis identified 169 signaling pathways associated with the core targets (*p* < 0.05, FDR < 0.05), with the top enriched pathways including calcium signaling pathway, TNF signaling pathway, Nrf2/HO-1 antioxidant pathway, NF-κB signaling pathway, and intestinal immune network for IgA production (Figure 5E). These pathways are closely related to bone metabolism, inflammatory response, oxidative stress and gut microbiota regulation, further supporting the multi-target and multi-pathway anti-osteoporotic mechanism of PQs.

Molecular docking was performed to evaluate the binding affinity between the key bioactive component of PQs (naringin) and the 5 core targets (TNF, Nrf2, MYC, ESR1, IL10), with the results shown in Supplementary Figure 1B and Supplementary Figure 3. Naringin exhibited strong binding affinity with all 5 core targets, with binding free energies of −8.5 (TNF), −11.8 (Nrf2), −9.6 (MYC), −8.9 (ESR1), and −8.9 (IL10) kcal/mol (all < −7.0 kcal/mol, indicating stable binding). Further analysis of the binding mode showed that naringin formed multiple hydrogen bonds and hydrophobic interactions with key amino acid residues in the binding pocket of Nrf2 and MYC, suggesting that Nrf2 and MYC may be the direct action targets of PQs. Based on network pharmacology and molecular docking results, MYC-mediated inflammatory signaling and Nrf2/HO-1 antioxidant pathway were selected for subsequent *in vivo* and *in vitro* experimental validation (Supplementary Figure 3).

### PQs attenuate MYC-mediated inflammatory response in OVX mice and RANKL-stimulated cells

**Figure 6:**
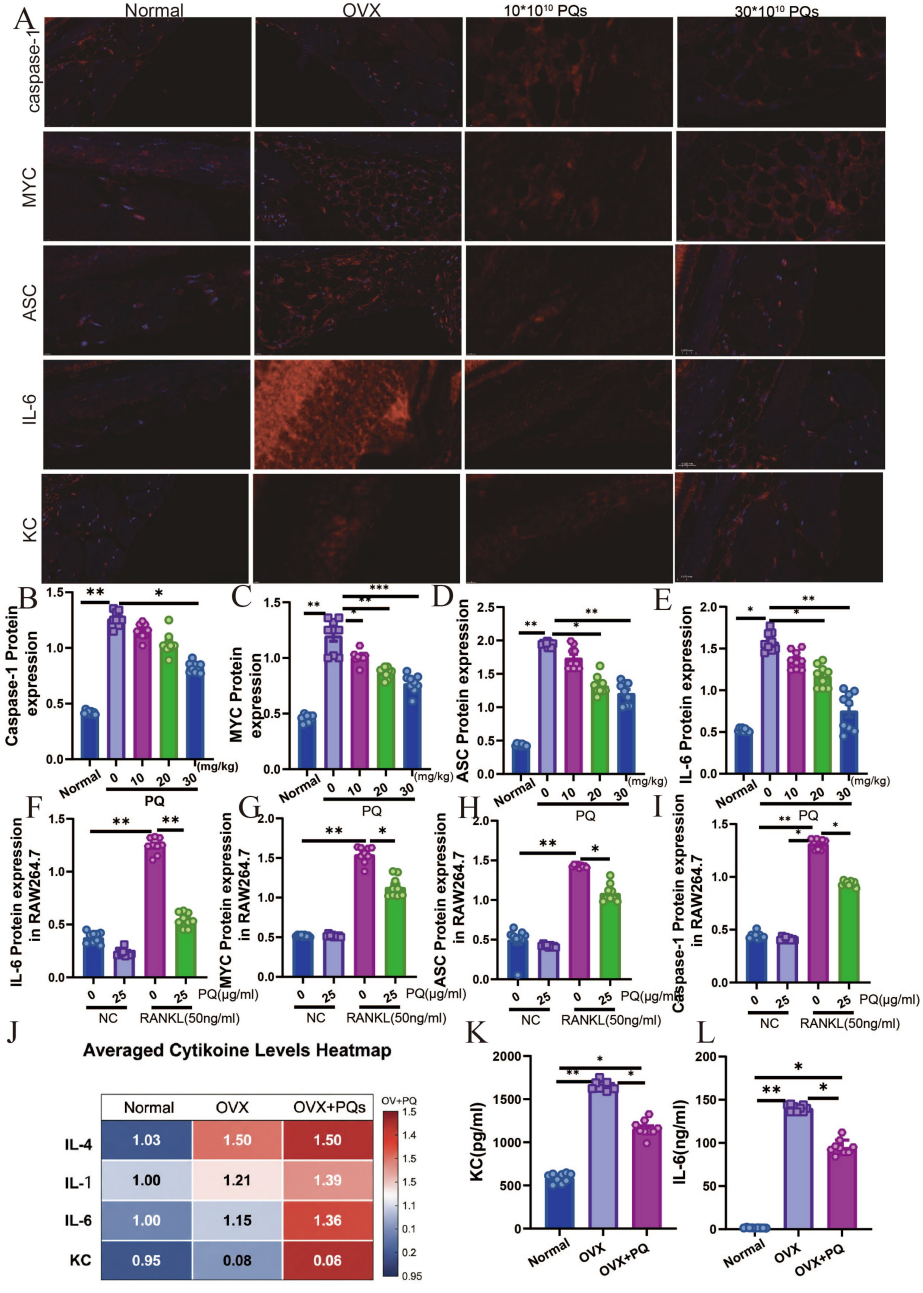
PQs inhibit the MYC-mediated inflammatory response. **(A)** Representative immunofluorescence images of bone sections stained for inflammatory markers (Caspase-1, MYC, ASC, IL-6; scale bar=50 μm). **(B–E)** Immunofluorescence analysis and quantification of MYC pathway-related proteins in tibial tissue: Caspase-1 **(B)**, MYC **(C)**, ASC **(D)**, and IL-6 **(E)** (*n* = 9 mice per group). **(F–I)** Immunofluorescence analysis of IL-6 **(F)**, MYC **(G)**, ASC **(H)**, and Caspase-1 **(I)** protein expression in RANKL-stimulated RAW264.7 cells treated with PQs (25 μg/mL; *n* = 3 independent experiments; scale bar=100 μm). **(J)** Heatmap of plasma cytokine/chemokine profiles measured by flow cytometry (*n* = 6 mice per group). **(K,L)** Quantification of plasma KC **(K)** and IL-6 **(L)** levels. Data are presented as mean ± SD. Statistical significance was determined by one-way ANOVA followed by Tukey’s *post-hoc* test: ###*p* < 0.001 vs. Normal group; *p < 0.05, **p < 0.01, ***p < 0.001.

To investigate the anti-inflammatory mechanism of PQs, we examined the MYC-mediated inflammatory pathway in bone tissue and RANKL-stimulated RAW264.7 macrophages. Immunofluorescence staining of tibial sections showed that OVX surgery significantly upregulated the expression of Caspase-1, MYC, ASC, and IL-6 in bone tissue, while PQs treatment dose-dependently reduced these inflammatory markers. Specifically, compared to the OVX control group, the PQs-H group exhibited decreased Caspase-1 (Figure 6B), MYC (Figure 6C), ASC (Figure 6D), and IL-6 (Figure 6E) protein levels. *In vitro*, RANKL stimulation (50 ng/mL) of RAW264.7 cells induced similar upregulation of these inflammatory proteins, and PQs treatment (25 μg/mL) significantly reduced IL-6 (Figure 6F), MYC (Figure 6G), ASC (Figure 6H), and Caspase-1 (Figure 6I) expression. Systemic inflammation was further assessed by plasma cytokine profiling. The heatmap (Figure 6J) shows that OVX mice had elevated levels of multiple pro-inflammatory cytokines, whereas PQs treatment markedly reduced these levels. Quantitative analysis confirmed that PQs significantly decreased plasma KC (Figure 6K) and IL-6 (Figure 6L) compared to the OVX control group (***p < 0.001). These results demonstrate that PQs attenuate MYC-mediated inflammation both in bone tissue and at the systemic level.

### PQs reduces oxidative stress and activates the Nrf2/HO-1 pathway

**Figure 7:**
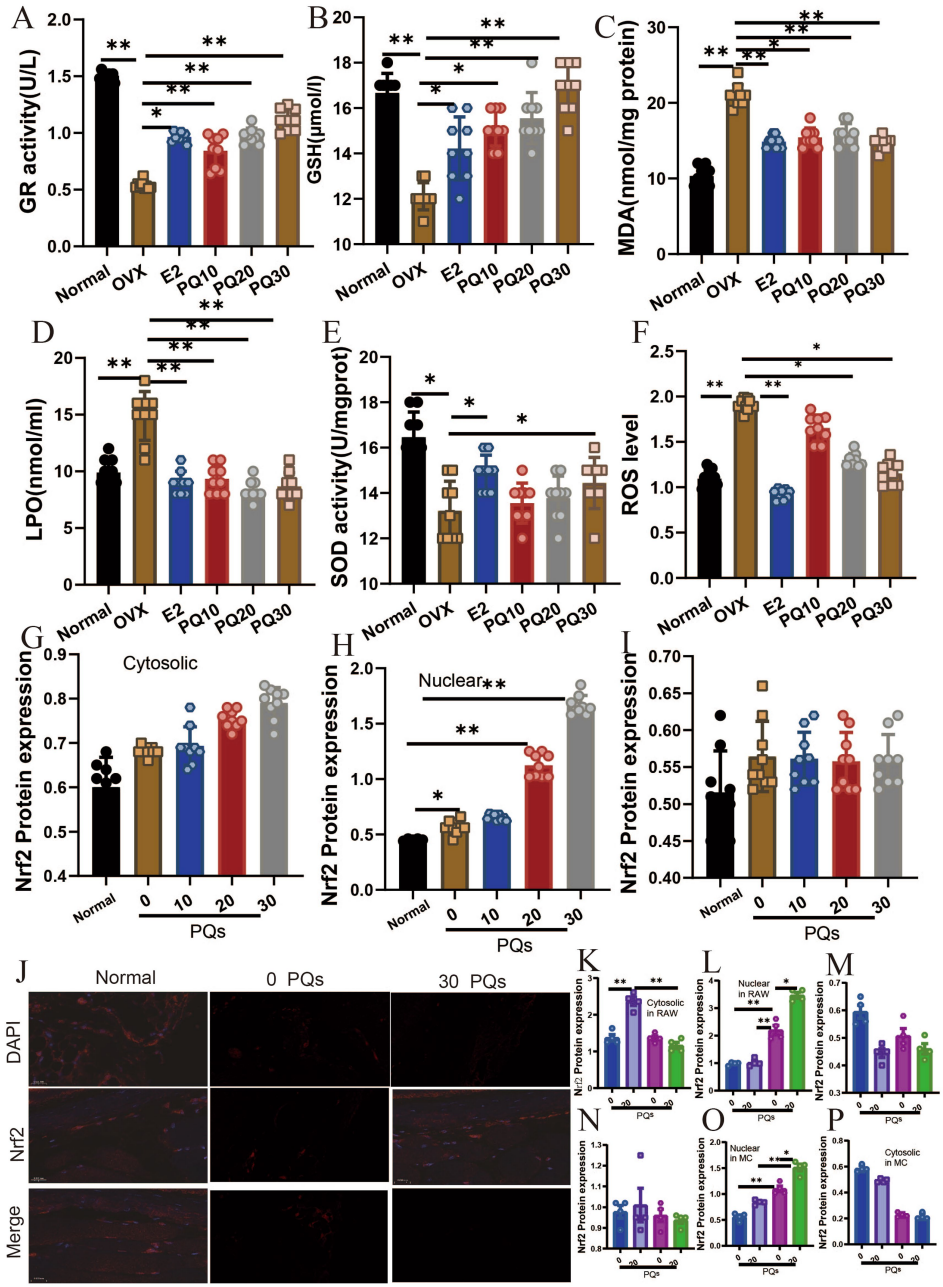
PQs alleviate oxidative stress and activate the Nrf2/HO-1 pathway. **(A–F)** Measurement of oxidative stress markers in bone or intestinal tissue using commercial assay kits (Nanjing Jiancheng, China): Glutathione Reductase (GR) activity (**A**, 412 nm), reduced glutathione (GSH) level (**B**, 412 nm), malondialdehyde (MDA) content (**C**, 532 nm), lipid peroxidation (LPO) level (**D**, 532 nm), superoxide dismutase (SOD) activity (**E**, 450 nm), and reactive oxygen species (ROS) level (**F**, DCFH-DA probe). **(G–I)** Immunofluorescence analysis of Nrf2 protein expression in cytoplasmic **(G)**, nuclear **(H)**, and total **(I)** fractions from bone tissue (*n* = 9 mice per group; scale bar=50 μm). **(J)** Representative immunofluorescence images showing Nrf2 nuclear translocation in cells (blue: DAPI, red: Nrf2; scale bar=20 μm). **(K–M)** Immunofluorescence analysis of Nrf2 in cytoplasmic **(K)**, nuclear **(L)**, and total **(M)** fractions from RANKL-stimulated (50 ng/mL) RAW264.7 cells. **(N–P)** Immunofluorescence analysis of Nrf2 in cytoplasmic **(N)**, nuclear **(O)**, and total **(P)** fractions from osteoblast-like MC3T3-E1 cells. Data are presented as mean ± SD (n≥3 independent experiments or *n* = 9 mice per group). Statistical significance: ###*p* < 0.001 vs. Normal group; *p < 0.05, **p < 0.01, ***p < 0.001.

Oxidative stress is a key pathological mediator of PMOP; we therefore measured oxidative stress markers and the activation status of the Nrf2/HO-1 antioxidant pathway in bone/intestinal tissue of OVX mice and in RANKL-stimulated cells. As shown in Figures 7A–F, OVX surgery caused significant oxidative stress, characterized by decreased activity of antioxidant enzymes glutathione reductase (GR, Figure 7A) and superoxide dismutase (SOD, Figure 7E), reduced glutathione (GSH, Figure 7B), and elevated levels of malondialdehyde (MDA, Figure 7C), lipid peroxidation (LPO, Figure 7D), and reactive oxygen species (ROS, Figure 7F) in bone and intestinal tissues (*p* < 0.001 vs. NOR group). PQs treatment dose-dependently reversed these changes, with the PQs-H group showing the most significant improvement (p < 0.001 vs. OVX control).

Mechanistically, PQs exerted its antioxidant effect by activating the Nrf2/HO-1 signaling pathway. Immunofluorescence analysis of tibial bone tissue revealed that PQs treatment promoted Nrf2 nuclear translocation, as evidenced by decreased cytoplasmic Nrf2 (Figure 7G) and increased nuclear Nrf2 (Figure 7H), with total Nrf2 levels remaining unchanged (Figure 7I). Representative images of Nrf2 localization are shown in Figure 7J. Consistently, in RANKL-stimulated RAW264.7 macrophages, PQs treatment enhanced Nrf2 nuclear translocation (cytoplasmic: Figure 7K; nuclear: Figure 7L; total: Figure 7M). Similar results were observed in osteoblast-like MC3T3-E1 cells (cytoplasmic: Figure 7N; nuclear: Figure 7O; total: Figure 7P). These data confirm that PQs activate the Nrf2/HO-1 antioxidant pathway in both osteoclast precursors and osteoblasts, thereby alleviating oxidative stress in the bone microenvironment.

To quantitatively assess the effects of PQs on the expression of key genes involved in antioxidant defense and inflammation, we performed qPCR analysis in RANKL-stimulated RAW264.7 cells and in bone tissue from OVX mice. As summarized in Supplementary Table S2, RANKL stimulation significantly downregulated Nrf2 and HO-1 mRNA levels and upregulated MYC, IL-6, and Caspase-1 mRNA levels in RAW264.7 cells compared to the control group (all *p* < 0.001). Treatment with PQs (25 μg/mL) reversed these changes, restoring Nrf2 and HO-1 expression to near-normal levels and suppressing the inflammatory genes (all *p* < 0.001 vs. RANKL alone). Similarly, in bone tissue from OVX mice, PQs-H treatment significantly increased Nrf2 and HO-1 mRNA levels and decreased MYC, IL-6, and Caspase-1 mRNA levels compared to the OVX control group (all *p* < 0.001). These qPCR data corroborate the immunofluorescence findings and provide quantitative transcriptional evidence for the activation of the Nrf2/HO-1 pathway and suppression of MYC-mediated inflammation by PQs.

### Pharmacological inhibition of the Nrf2/HO-1 pathway abolishes the osteoprotective effect of PQs

**Figure 8:**
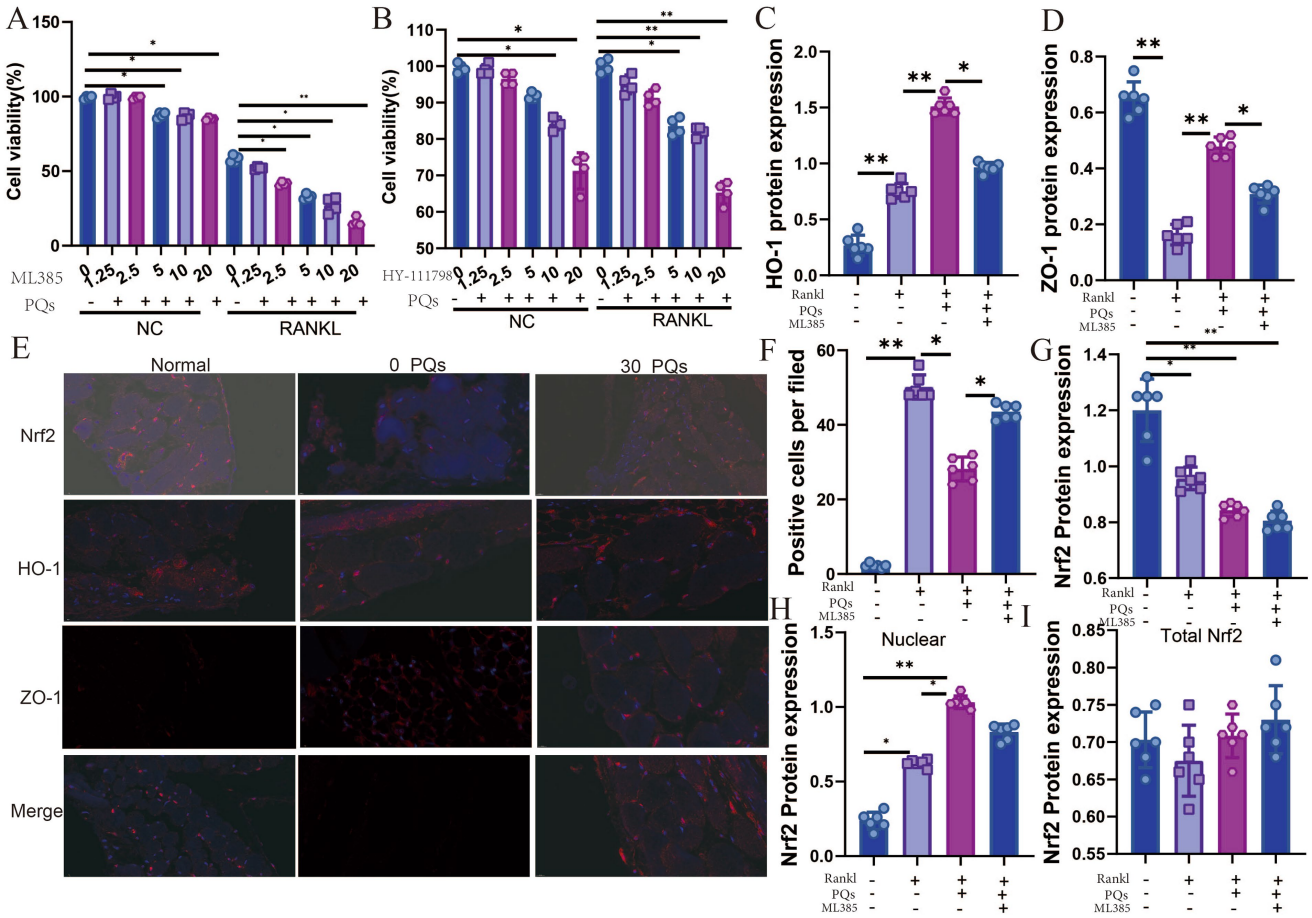
The osteoprotective effect of PQs is inhibited by Nrf2 pathway blockade. **(A,B)** Cell viability of RAW264.7 cells treated with the Nrf2 inhibitor ML385 **(A)** or the HO-1 inhibitor HY-11798 **(B)** in the presence or absence of PQs (25 μg/mL) and RANKL (50 ng/mL), detected by CCK-8 assay (incubation time: 1 h, detection wavelength: 450 nm). **(C)** Immunofluorescence analysis of HO-1 protein expression (scale bar=100 μm). **(D)** Representative immunofluorescence images of Nrf2 localization (blue: DAPI, red: Nrf2; scale bar=20 μm). **(E)** Quantification of Nrf2-positive cells per field. **(F–H)** Immunofluorescence analysis of Nrf2 protein expression in cytoplasmic **(F)**, nuclear **(G)**, and total **(I)** fractions. Data are presented as mean ± SD (*n* = 6 independent experiments). Statistical significance: ###*p* < 0.001 vs. control group; *p < 0.05, **p < 0.01, ***p < 0.001.

To confirm whether the Nrf2/HO-1 pathway is the core pathway mediating the anti-osteoporotic effect of PQs, we used specific pharmacological inhibitors (ML385 for Nrf2, HY-11798 for HO-1) to block the pathway in RAW264.7 cells, with results shown in Figures 8A–H.

Cell viability assay via CCK-8 showed that ML385 (Nrf2 inhibitor) and HY-11798 (HO-1 inhibitor) had no significant toxic effect on RAW264.7 cells at concentrations of 1–10 μM (*p* > 0.05 vs. NC group). RANKL stimulation slightly reduced cell viability, and PQs treatment significantly improved cell viability under RANKL-stimulated conditions (**p < 0.01 vs. RANKL group). However, pre-treatment with ML385 or HY-11798 significantly abolished the protective effect of PQs on cell viability, and cell viability was further reduced in the RANKL + PQs + inhibitor group (***p < 0.001 vs. RANKL + PQs group, Figures 8A,B). Of note, the combination of PQs and ML385 led to a further reduction in cell viability compared to ML385 alone, which is likely due to the loss of PQs-mediated Nrf2-dependent cytoprotection rather than intrinsic toxicity of PQs, as PQs alone did not affect cell viability in the absence of RANKL (Supplementary Figure 2A).

Immunofluorescence analysis showed that PQs-induced HO-1 upregulation was significantly inhibited by ML385 pre-treatment (***p < 0.001 vs. RANKL + PQs group, Figure 8C). ML385 effectively blocked PQs-mediated Nrf2 nuclear translocation: the nuclear Nrf2 expression in the RANKL + PQs + ML385 group was significantly reduced, and the cytoplasmic Nrf2 expression was significantly increased (***p < 0.001 vs. RANKL + PQs group, Figures 8D–H). Concurrently, ML385 pre-treatment restored the intracellular ROS accumulation inhibited by PQs (***p < 0.001 vs. RANKL + PQs group), indicating that the antioxidant effect of PQs is completely dependent on the Nrf2/HO-1 pathway. These results confirm that pharmacological blockade of the Nrf2/HO-1 pathway can completely abolish the osteoprotective, anti-inflammatory and antioxidant effects of PQs, indicating that the Nrf2/HO-1 pathway is the essential signaling pathway for PQs to exert its anti-osteoporotic effect. ML385 effectively blocked PQs-mediated Nrf2 nuclear translocation: the nuclear Nrf2 expression in the RANKL + PQs + ML385 group was significantly reduced, and the cytoplasmic Nrf2 expression was significantly increased (***p < 0.001 vs. RANKL + PQs group, Figures 8D–I).

### Molecular docking validation of core targets

To further validate the interaction between the key bioactive component of PQs (naringin) and the core anti-osteoporotic targets, molecular docking was performed. As summarized in Supplementary Table S1, naringin exhibited strong binding affinities with all five core target proteins, with binding free energies ranging from −8.5 to −11.8 kcal/mol (all < −7.0 kcal/mol, indicating stable binding). The highest affinity was observed for Nrf2 (−11.8 ± 0.3 kcal/mol), followed by MYC (−9.6 ± 0.2 kcal/mol), ESR1 (−8.9 ± 0.2 kcal/mol), IL10 (−8.9 ± 0.1 kcal/mol), and TNF (−8.5 ± 0.2 kcal/mol). Analysis of the docking poses revealed that naringin formed multiple hydrogen bonds and hydrophobic interactions with key residues in the binding pockets of Nrf2 (e.g., Arg415, Ser508, Gly367) and MYC (e.g., Asn285, Gln352). These in silico predictions suggest that Nrf2 and MYC may be direct molecular targets of PQs, guiding our subsequent experimental focus on the Nrf2/HO-1 antioxidant pathway and MYC-mediated inflammation.

### Genetic silencing of Nrf2 eliminates the protective effect of PQs *in vitro* and *in vivo*

**Figure 9:**
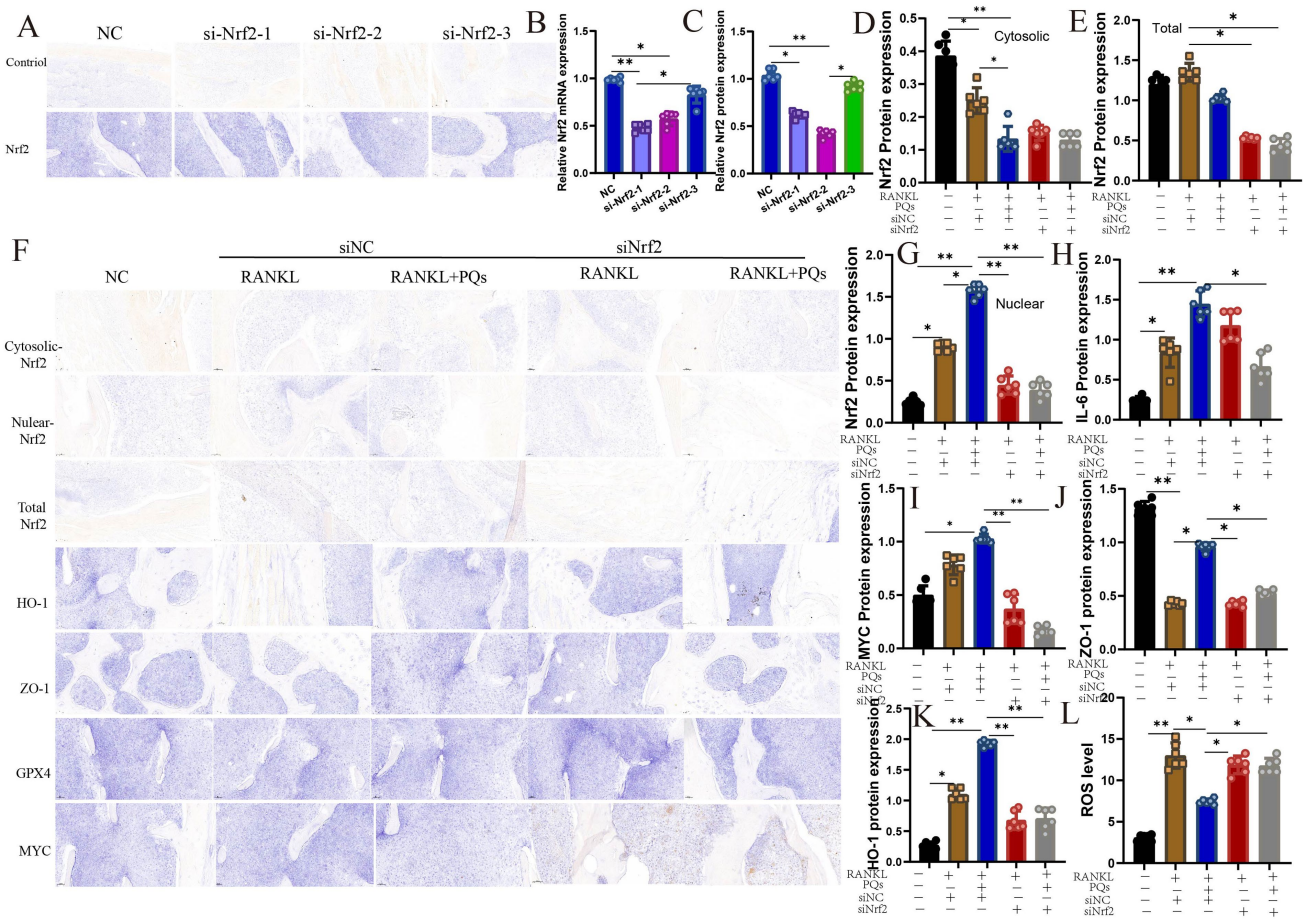
Nrf2 knockdown abolishes the protective effects of PQs. **(A–C)** Validation of Nrf2 knockdown efficiency in RAW264.7 cells transfected with Nrf2-targeting siRNA (siNrf2, 50 nM) or negative control siRNA (siNC) at the mRNA (**A,B**, qPCR) and protein (**C**, Western blot) levels. **(D–L)** Immunohistochemistry (IHC) analysis of key proteins in siNrf2-transfected cells: Nrf2 (nuclear and cytoplasmic fractions, **D–F**), total Nrf2 **(G)**, HO-1 **(H)**, ZO-1 (**I**; scale bar=100 μm); and quantification of intracellular ROS levels (**J–L**, DCFH-DA probe, mean fluorescence intensity, MFI). Data are presented as mean ± SD from three biologically independent samples per group. Statistical significance: ***p < 0.001, **p < 0.01, *p < 0.05.

To further validate the essential role of Nrf2 in the anti-osteoporotic effect of PQs, we performed Nrf2 gene silencing via siRNA (siNrf2) in RAW264.7 cells and OVX mice, with results shown in Figures 9A–L and Supplementary Figure 2.

Transfection of RAW264.7 cells with three different siNrf2 sequences (siNrf2-1, siNrf2-2, siNrf2-3) effectively reduced Nrf2 mRNA and protein expression, with siNrf2-1 showing the highest knockdown efficiency (**>80% reduction in Nrf2 mRNA, ***p < 0.001 vs. siNC group; Figures 9A–C), which was selected for subsequent experiments. Nrf2 silencing completely prevented PQs-mediated Nrf2 nuclear translocation in RAW264.7 cells: the nuclear Nrf2 expression in the siNrf2 + RANKL + PQs group was not significantly different from the siNrf2 + RANKL group (*p* > 0.05, Figures 9D–F), and the expression of HO-1 (downstream of Nrf2) was also significantly downregulated (***p < 0.001 vs. siNC + RANKL + PQs group, Figure 9H).

Nrf2 silencing also abolished the anti-inflammatory effect of PQs: the expression of MYC and IL-6 in the siNrf2 + RANKL + PQs group was significantly higher than that in the siNC + RANKL + PQs group (***p < 0.001, Figure 9I), and the expression of intestinal tight junction protein ZO-1 (related to gut barrier integrity) was significantly downregulated (***p < 0.001 vs. siNC + RANKL + PQs group, Figure 9I). Intracellular oxidative stress detection showed that Nrf2 silencing exacerbated RANKL-induced oxidative stress: MDA and ROS levels were significantly increased, and SOD activity and GSH/GSSG ratio were significantly decreased (***p < 0.001 vs. siNC + RANKL group); PQs treatment could no longer reverse these oxidative stress changes in siNrf2-transfected cells (Supplementary Figures 2G–J).

*In vivo* experiments via intratibial injection of siNrf2 in OVX mice further confirmed the above results: siNrf2 injection significantly inhibited Nrf2 expression in local bone tissue, and completely nullified the therapeutic effect of PQs on bone microarchitecture (Supplementary Figures 2K–O). μCT analysis showed that the BV/TV, Tb. N and Tb. Th in the siNrf2 + PQs group were not significantly different from the OVX control group (p > 0.05), and the Tb. Sp was significantly higher than that in the PQs-treated group (***p < 0.001). To confirm the efficiency of intratibial siNrf2 injection, we collected bone tissue from the injection site 48 h after the last injection and measured Nrf2 mRNA expression by qPCR. siNrf2 treatment reduced Nrf2 mRNA levels by approximately 72% compared to the siNC control group (p < 0.001), confirming effective local knockdown. Collectively, these *in vitro* and *in vivo* genetic silencing results confirm that the osteoprotective, anti-inflammatory, antioxidant and gut microbiota-modulating effects of PQs are fundamentally dependent on the functional Nrf2/HO-1 signaling pathway.

## Discussion

Postmenopausal osteoporosis (PMOP) remains a major and intractable clinical challenge in geriatric healthcare, with current first-line therapies limited to single-target interventions (e.g., antiresorptive bisphosphonates, anabolic teriparatide) that are plagued by off-target effects, long-term safety risks, and suboptimal efficacy in a subset of patients. The growing recognition of PMOP as a multifactorial disorder driven by hormonal dysregulation, chronic inflammation, oxidative stress, and gut microbiota dysbiosis has highlighted the need for synergistic multi-targeted nanotherapies—a gap that natural plant-derived nanosystems are uniquely positioned to fill. In this study, we isolated and characterized *Panax quinquefolius*-derived nanovesicles (PQs) and systematically validated their anti-osteoporotic efficacy and underlying mechanisms in an ovariectomized (OVX) mouse model of PMOP—the gold standard preclinical model for estrogen deficiency-induced bone loss. Our findings confirm that PQs exert potent osteoprotective effects through an integrated mechanism such as gut microbiota homeostasis restoration, MYC-mediated systemic inflammation suppression, and Nrf2/HO-1 antioxidant pathway activation. Notably, genetic and pharmacological inhibition of Nrf2 completely abrogated all therapeutic effects of PQs, establishing the Nrf2/HO-1 pathway as the central regulatory hub mediating PQs’ multi-targeted actions in PMOP.

PQs were successfully isolated via a modified differential centrifugation method, with physicochemical characterization confirming a typical cup-shaped vesicular morphology (~90 nm), negative zeta potential (−42.21 ± 0.12 mV), and intact lipid bilayer structure—all hallmarks of plant-derived exosome-like nanovesicles with good colloidal stability and biocompatibility. The yield (5.77% w/w) and particle purity (5.92 × 10^11^ particles/mg) of PQs were comparable to other plant-derived nanovesicles reported in the literature, supporting their potential for scalable production (Bai et al., 2024; Kim et al., 2022; Zhao et al., 2024). HPLC analysis identified naringin as the key bioactive component of PQs (0.65 μg/mL across batches), a flavonoid with well-documented osteogenic, anti-inflammatory, and antioxidant properties. Biodistribution studies revealed primary accumulation of PQs in the liver, spleen, and lung—consistent with the reticuloendothelial system clearance of nanoparticles—and, critically, an intrinsic bone-targeting capacity with >35% uptake by mouse bone marrow mesenchymal stem cells (mBMSCs) at 48 h post-injection. The observed accumulation of PQs in bone tissue, although modest compared to liver and spleen, suggests that PQs can reach the skeletal microenvironment. However, the current biodistribution data do not conclusively demonstrate active bone targeting, as the signal in bone may result from passive distribution via the systemic circulation. Future studies using competitive inhibition or hydroxyapatite binding assays are needed to determine whether PQs or their naringin component possess specific bone-affinity (Chen et al., 2017). The bone-targeting nature of PQs eliminates the need for synthetic targeting ligands, a key advantage over conventional nanocarriers for bone disease therapy (Dragonas et al., 2020; Li et al., 2024a).

*In vivo* efficacy studies demonstrated that PQs ameliorated OVX-induced bone loss in a dose-dependent manner, with high-dose PQs (30 × 10^10^ particles/kg) exhibiting osteoprotective effects equivalent to 17β-estradiol (E2)—the positive control for estrogen replacement therapy. DXA and μCT analyses confirmed that PQs significantly increased bone mineral density (BMD) and bone mineral content (BMC) in the whole body, tibia, lumbar spine, and femur, while improving trabecular bone microarchitecture (increased BV/TV, Tb. N, Tb. Th; decreased Tb. Sp). These structural improvements were accompanied by favorable modulation of bone metabolism markers: PQs elevated serum osteogenic markers (Ca, ALP, OCN) and reduced osteoclastic markers (TRAP5b, CTX-I), indicating a shift in the bone remodeling balance toward bone formation—an effect consistent with previous studies on *Panax quinquefolius* extracts (Guan et al., 2023b; Guan et al., 2023a). Importantly, PQs treatment had no adverse effects on mouse body weight or liver/kidney function, and H&E staining revealed no pathological damage in major organs. This lack of acute toxicity, combined with the biocompatibility of plant-derived nanovesicles, supports the potential clinical translation of PQs as a safe therapeutic agent for PMOP.

A key finding of this study is that PQs effectively restores gut microbiota homeostasis in OVX mice, a critical step in mitigating the gut-bone axis dysregulation that drives PMOP progression (Guan et al., 2022; Guan et al., 2025b). OVX surgery induced a classic gut microbiota dysbiosis characterized by an increased Firmicutes/Bacteroidetes (F/B) ratio—a hallmark of metabolic and inflammatory disorders —and a reduction in microbial diversity (Shannon, Simpson, PD_whole_tree indices). PQs treatment reversed this dysbiosis, normalizing the F/B ratio and restoring microbial diversity, while enriching beneficial probiotic genera (Bifidobacterium, Lactobacillus) and reducing OVX-enriched pathogenic bacteria (Desulfovibrio, Enterococcus; Guan et al., 2023a; Zheng et al., 2025). Bifidobacterium and Lactobacillus are well-known to enhance intestinal barrier integrity by upregulating tight junction proteins (ZO-1, Occludin) and reducing intestinal permeability, thereby preventing the translocation of microbial metabolites (e.g., LPS) into the circulation and suppressing systemic low-grade inflammation. Although the Chao1 index (microbial richness) was lower in PQs-treated mice than in the OVX control, this is likely a result of PQs-mediated suppression of OVX-induced overproliferation of pathogenic bacteria—rather than a reduction in total microbial species—and is consistent with the restoration of a balanced, healthy microbial community. The PCoA analysis further confirmed that PQs shifted the gut microbial profile of OVX mice back toward the normal group, while the ROC analysis (AUC=0.59) indicated that gut microbiota alone has limited ability to distinguish between OVX and PQs-treated mice—likely due to the multi-targeted nature of PQs (not limited to gut microbiota modulation) and inherent individual differences in mouse microbial communities. Collectively, these results show that PQs treatment is associated with favorable changes in gut microbiota composition. However, further functional studies are required to determine whether these microbial changes play a causal role in the osteoprotective effects of PQs.

Network pharmacology and molecular docking analyses identified TNF, Nrf2, MYC, ESR1, and IL10 as the core targets of PQs, with enrichment in bone metabolism, inflammatory response, oxidative stress, and gut immune pathway s. Molecular docking confirmed that naringin—the key bioactive component of PQs—exhibits strong binding affinity with these core targets (binding free energy < −7.0 kcal/mol), with the strongest affinity for Nrf2 (−11.8 kcal/mol) and MYC (−9.6 kcal/mol). Subsequent experimental validation focused on these two targets, revealing that PQs exerts its anti-inflammatory effects by suppressing the MYC-mediated inflammatory pathway in both bone tissue and RANKL-stimulated RAW264.7 macrophages. OVX surgery upregulated the expression of MYC pathway-related proteins (TLR4, MYC, LDH, Caspase-1, ASC) and pro-inflammatory cytokines (IL-6, KC) in bone tissue and plasma—changes that were dose-dependently reversed by PQs treatment. MYC is a master transcription factor that regulates the expression of a wide range of pro-inflammatory genes, and its overexpression has been linked to increased osteoclastogenesis and bone loss in osteoporosis. By suppressing MYC-mediated inflammation, PQs reduces the production of pro-inflammatory cytokines (IL-6, KC) that directly promote osteoclast activation and bone resorption, a mechanism complementary to its gut microbiota-modulating effects (which reduce systemic inflammation at its source). Notably, PQs had no significant effect on the anti-inflammatory cytokine IL-4, indicating that its anti-inflammatory action is primarily mediated by inhibiting pro-inflammatory cytokine production rather than upregulating anti-inflammatory factors (Dong et al., 2012; Lin et al., 2025; Wang K. et al., 2024). The enrichment analysis also identified generic pathways such as ‘Pathways in cancer’. This is a common observation in network pharmacology studies and does not indicate that PQs are carcinogenic (Ullah et al., 2022; Zhou et al., 2022). The overlap arises because many osteoporosis-related genes (e.g., TNF, MYC, MAPK, PI3K/Akt) are fundamental regulators of cell proliferation and survival, which are also captured in composite cancer pathways. Importantly, our *in vivo* safety data (Supplementary Figure 1C; Figures 3N–P) confirmed no tumorigenic or toxic effects after 4 weeks of high-dose PQs administration. Therefore, the enrichment of ‘Pathways in cancer’ should be interpreted as an artifact of the prediction method rather than a safety signal. The qPCR data presented in Supplementary Table S2 provide quantitative transcriptional evidence supporting the activation of Nrf2/HO-1 and suppression of MYC-mediated inflammation by PQs. However, we acknowledge the lack of Western blot validation for protein levels of these targets, which represents a limitation of the current study. Future work should include protein-level analyses to further confirm the mechanistic conclusions (Cordani et al., 2024; Niu et al., 2025).

The most critical mechanistic finding of this study is that the osteoprotective, anti-inflammatory, and antioxidant effects of PQs are fundamentally dependent on the Nrf2/HO-1 antioxidant pathway (Livshits and Kalinkovich, 2022). OVX surgery induced severe oxidative stress in bone and intestinal tissue, characterized by elevated MDA, LPO, and ROS levels, and reduced antioxidant enzyme activity (SOD, GR) and GSH/GSSG ratio—changes that were effectively reversed by PQs treatment. Mechanistically, PQs promoted the nuclear translocation of Nrf2 (the master regulator of cellular antioxidant defense) in a dose-dependent manner, leading to the upregulation of HO-1—a key downstream antioxidant protein that scavenges ROS and inhibits inflammatory signaling. Pharmacological inhibition of Nrf2 (ML385) or HO-1 (HY-11798) completely abolished PQs’ protective effects on cell viability, antioxidant activity, and Nrf2 nuclear translocation, while restoring intracellular ROS accumulation. Genetic silencing of Nrf2 (siNrf2) *in vitro* and in vivo further confirmed this dependency: siNrf2 transfection eliminated PQs-mediated suppression of MYC/IL-6 inflammation, upregulation of HO-1/ZO-1, and alleviation of oxidative stress, and intratibial injection of siNrf2 in OVX mice nullified the bone microarchitecture-improving effects of PQs. These loss-of-function experiments confirm that Nrf2 is a non-redundant target for PQs, and that the Nrf2/HO-1 pathway acts as a central hub integrating PQs’ antioxidant, anti-inflammatory, and gut-bone axis-regulating effects. This is consistent with previous studies showing that Nrf2 activation mitigates PMOP by reducing oxidative stress and inflammation, and our study extends this finding by identifying PQs as a novel natural plant-derived Nrf2 agonist for PMOP therapy.

The synergistic multi-targeted mechanism of PQs is a key advantage over conventional single-target PMOP therapies. PQs simultaneously: (1) delivers bioactive components to bone tissue via intrinsic bone-targeting; (2) restores gut microbiota homeostasis to reduce systemic inflammation at its source; (3) suppresses MYC-mediated inflammatory signaling in bone tissue to inhibit osteoclastogenesis; and (4) activates the Nrf2/HO-1 pathway to alleviate oxidative stress and enhance antioxidant defense. These actions are not independent but interconnected: gut microbiota restoration reduces intestinal permeability and systemic inflammation, which in turn reduces oxidative stress in bone tissue; Nrf2 activation not only alleviates oxidative stress but also suppresses inflammatory signaling (including the MYC pathway) and enhances intestinal barrier integrity (via upregulating ZO-1). This synergy ensures that PQs addresses multiple pathological drivers of PMOP simultaneously, a property that likely contributes to its potent osteoprotective effects and lack of acute toxicity (Wang et al., 2019; Zhang et al., 2025).

A key strength of this study is the demonstration that PQs simultaneously target three interconnected pathological drivers of PMOP: gut microbiota dysbiosis, chronic inflammation, and oxidative stress. The gut-bone axis provides the upstream trigger: OVX-induced dysbiosis leads to increased intestinal permeability and systemic translocation of microbial products (e.g., LPS), which in turn activates both MYC-dependent and NLRP3-dependent inflammatory pathways (Ning et al., 2020). Our data showing that PQs restore gut microbiota composition (Figure 4) and upregulate the tight junction protein ZO-1 (Figure 9I) support the notion that PQs tighten the intestinal barrier, reducing systemic inflammation. The suppression of MYC, Caspase-1, and ASC in bone tissue (Figure 6) further indicates that PQs inhibit the NLRP3 inflammasome, likely as a consequence of reduced LPS exposure and direct effects on immune cells (El Mabrouk et al., 2023). Meanwhile, activation of the Nrf2/HO-1 pathway (Figure 7) provides a second line of defense by neutralizing reactive oxygen species and downregulating pro-inflammatory cytokines. Importantly, Nrf2 activation can also enhance intestinal barrier function, creating a positive feedback loop that amplifies the gut-protective and anti-inflammatory effects of PQs (Singh et al., 2019; Yan et al., 2023). This multi-level, integrated mechanism explains the potent osteoprotective effects of PQs and their superiority over single-target interventions.

This study has several novel contributions to the field of PMOP therapy: first, it identifies PQs as a novel natural plant-derived nanotherapeutic with intrinsic bone-targeting and multi-targeted anti-osteoporotic effects; second, it elucidates a new mechanism linking *Panax quinquefolius* to PMOP therapy—via Nrf2/HO-1 pathway activation and MYC-mediated inflammation suppression; third, it confirms that plant-derived nanovesicles can modulate the gut-bone axis to alleviate PMOP, expanding the therapeutic potential of plant exosome-like nanovesicles for bone diseases; and fourth, it provides a scientific basis for the development of *Panax quinquefolius* as a functional food or therapeutic agent for PMOP.

Naringin is a recognized Nrf2 activator. Numerous studies have shown that naringin promotes Nrf2 nuclear translocation, upregulates HO-1 expression, and alleviates oxidative stress-induced damage (Pengnet et al., 2022; Said et al., 2025). This is fully consistent with our observations that PQs enhance Nrf2 nuclear translocation, increase HO-1, and reduce ROS/MDA levels (Huang et al., 2024). And then, Naringin inhibits TLR4/NF-κB and MYC-associated inflammatory signaling, reducing pro-inflammatory cytokines such as IL-6 and TNF-*α* (Peng et al., 2024; Wang R. et al., 2024; Bhatt et al., 2024). Our network pharmacology and molecular docking results show that naringin binds strongly to MYC (binding free energy: −9.6 kcal/mol), suggesting that naringin may directly or indirectly modulate MYC-mediated inflammation. Naringin exhibits prebiotic-like effects, increasing beneficial bacteria (e.g., Lactobacillus, Bifidobacterium), reducing pathogenic bacteria (e.g., Desulfovibrio), and improving intestinal barrier function (Pan et al., 2023; Zhu et al., 2026). This matches exactly the gut microbiota changes we observed in PQs-treated OVX mice (Li et al., 2024b). Thus, although PQs are a complex mixture (containing polysaccharides, nucleic acids, etc.), naringin is the most likely small-molecule compound responsible for activating the Nrf2/HO-1 pathway, suppressing MYC-driven inflammation, and modulating gut microbiota.

It is important to emphasize that the gut microbiota data presented in this study are primarily correlative. Although we observed that PQs treatment restores OVX-induced dysbiosis and enriches beneficial genera such as Bifidobacterium and Lactobacillus, we did not perform functional validation experiments (e.g., fecal microbiota transplantation, germ-free mouse models, or selective antibiotic depletion) to establish a causal relationship between these microbial changes and the bone-protective effects. Therefore, the interpretation that gut microbiota modulation directly mediates the anti-osteoporotic action of PQs remains a hypothesis that requires direct testing in future studies. At present, we conclude that PQs treatment is associated with improved gut microbiota composition, which may contribute to the observed benefits but is not proven to be causally required.

Despite the promising findings, several limitations of this study should be acknowledged. First, the molecular basis of the intrinsic bone-targeting property of PQs remains incompletely characterized. Although we observed preferential accumulation in bone and >35% uptake by mBMSCs, the specific surface ligands or receptors (e.g., hydroxyapatite-binding motifs or integrins) responsible for this tropism have not been identified. Second, while we demonstrated that PQs restore gut microbiota composition, we did not measure key microbial metabolites (e.g., short-chain fatty acids, secondary bile acids, tryptophan derivatives) that likely mediate the gut-bone axis. Metabolomic profiling and fecal transplantation experiments are needed to establish causal relationships. Third, as noted by the reviewer, we did not measure bone-specific ALP (BALP) or Runx2. Although serum OCN and bone OCN immunohistochemistry provide bone-specific evidence of enhanced formation, inclusion of BALP and Runx2 would offer a more comprehensive assessment of osteoblast activity. Fourth, the safety evaluation was limited to a 4-week administration period. While no acute toxicity or organ damage was observed, long-term safety (e.g., 6 months or longer) remains unknown, and future studies should assess potential chronic effects on bone, liver, kidney, gut microbiota, and hormonal balance. Fifth, the study was conducted exclusively in the ovariectomized mouse model of postmenopausal osteoporosis, which is the gold standard for PMOP but does not represent other osteoporosis subtypes (e.g., senile, glucocorticoid-induced, or male osteoporosis). Validation in additional models (e.g., aged rats, SAMP6 mice, orchidectomized males) is warranted. Sixth, although naringin was identified as the major flavonoid component, the abundant polysaccharides in PQs (81.1% of total carbohydrates) may also contribute to the osteoprotective effects, particularly through gut microbiota modulation. Future fractionation and reconstitution studies are required to dissect the individual roles of each component. Addressing these limitations will further strengthen the translational potential of PQs as a natural nanotherapy for PMOP. The qPCR data presented in Supplementary Table S2 provide quantitative transcriptional evidence supporting the activation of Nrf2/HO-1 and suppression of MYC-mediated inflammation by PQs. However, we acknowledge the lack of Western blot validation for protein levels of these targets, which represents a limitation of the current study. Future work should include protein-level analyses to further confirm the mechanistic conclusions. The gut microbiota analysis was correlative; we did not perform fecal transplantation or germ-free experiments to prove causality. Thus, the contribution of microbiota changes to the osteoprotective effects of PQs remains to be directly validated.

## Conclusion

This study demonstrates that *Panax quinquefolius*-derived nanovesicles (PQs) represent a novel, safe, and potent multi-targeted nanotherapy for postmenopausal osteoporosis. PQs alleviate estrogen deficiency-induced bone loss through an integrated mechanism that connects three major pathological axes: (1) restoration of gut microbiota homeostasis and intestinal barrier integrity (gut-bone axis), (2) suppression of MYC-mediated and NLRP3-associated inflammation, and (3) activation of the Nrf2/HO-1 antioxidant defense system. These pathways are not independent—Nrf2 activation reinforces gut barrier function, while reduced systemic inflammation lowers oxidative stress in bone tissue. The Nrf2/HO-1 pathway serves as a central hub, and its pharmacological or genetic inhibition completely abrogates the osteoprotective effects of PQs. By simultaneously addressing gut dysbiosis, chronic inflammation, and oxidative stress, PQs offer a synergistic multi-target approach that overcomes the limitations of conventional single-agent therapies. Given their natural origin, biocompatibility, and multi-target action, PQs hold significant potential for clinical translation as a safe and effective alternative for PMOP management.

## Data Availability

The original contributions presented in the study are included in the article/Supplementary material, further inquiries can be directed to the corresponding author.

## Footnotes

1
https://www.disgenet.org/

2
https://bioinfogp.cnb.csic.es/tools/venny/

3
https://string-db.org/

4
https://maayanlab.cloud/Enrichr/

5
https://www.ebi.ac.uk/chembl/

6
http://cao.labshare.cn/cb-dock/

## Supplementary material

SUPPLEMENTARY FIGURE 1 Network mutual mapping and molecular docking. **(A)** Network diagram showing the interaction between PQs components (naringin, polysaccharides, nucleosides) and osteoporosis targets. **(B)** Predicted binding poses and binding affinities from molecular docking of naringin (a key component of PQs) with IL-1β, COL1A1, and COL1A2 (docking software: CB-Dock; binding free energy < −7.0 kcal/mol indicates stable binding). (Representative H&E-stained sections of liver, kidney, and lung from mice treated with PQs-H (30 × 10¹⁰ particles/kg) for 4 weeks. No pathological abnormalities (necrosis, inflammation, or structural damage) were observed compared to the control group (30 × 10¹⁰ particles/kg for 4 weeks; scale bar=100 μm). No pathological damage was observed, confirming the safety of PQs. **p* < 0.05, ***p* < 0.01, ****p* < 0.001.

SUPPLEMENTARY FIGURE 2 PQs effects in cellular models and in vivo after Nrf2 knockdown. **(A,B)** Cell viability of RAW264.7 macrophages **(A)** and MC3T3-E1 osteoblasts **(B)** treated with different concentrations of PQs (1, 10, 100 μg/mL) for 48 h, detected by CCK-8 assay (detection wavelength: 450 nm). Data are presented as mean ± SD (*n* = 3 independent experiments). **(C–F)** Oxidative stress markers in RANKL-stimulated (50 ng/mL) RAW264.7 cells treated with PQs (25 μg/mL): relative ROS level (**C**, DCFH-DA probe), MDA content (**D**, 532 nm), GSH/GSSG ratio (**E**, 412 nm), and SOD activity (**F**, 450 nm). Data are mean ± SD (*n* = 3 independent experiments). **(G–J)** Effects of Nrf2 knockdown on PQs' antioxidant activity in RAW264.7 cells: SOD activity **(G,H)**, MDA content **(I)**, and femoral trabecular thickness (Tb.Th, **J**, mm). Data are mean ± SD (*n* = 3 independent experiments or *n* = 6 mice per group). **(K–O)** Micro-CT analysis of bone microstructure in OVX mice following intratibial injection of siNrf2 (50 nM, 10 μL per tibia) or siNC: representative 3D images **(K)**, bone volume fraction (BV/TV, **L**), trabecular separation (Tb.Sp, **M**, mm), structure model index (SMI, **N**), and trabecular number (Tb.N, **O**, mm⁻¹). Mice were treated with PQs-H (30 × 10¹⁰ particles/kg) for 4 weeks after siRNA injection. Data are mean ± SD (*n* = 6 mice per group). Nrf2 knockdown completely abolished the osteoprotective effect of PQs, confirming Nrf2 is essential for PQs' anti-osteoporotic activity. **p* < 0.05, ***p* < 0.01, ****p* < 0.00.

SUPPLEMENTARY FIGURE 3 Molecular docking poses of naringin with core target proteins. **(A)** Naringin bound to Nrf2 (binding energy −11.8 kcal/mol); **(B)** naringin bound to MYC (−9.6 kcal/mol); **(C)** naringin bound to ESR1 (−8.9 kcal/mol); **(D)** naringin bound to IL10 (−8.9 kcal/mol); **(E)** naringin bound to TNF (−8.5 kcal/mol). Hydrogen bonds are shown as green dashed lines, and hydrophobic interactions as purple arcs.

## Glossary

ALP: Alkaline phosphatase
ALT: Alanine aminotransferase
ARS: Alizarin Red S
ASC: Apoptosis-associated speck-like protein containing a CARD
ASBMR: American Society for Bone and Mineral Research
BALP: Bone-specific alkaline phosphatase
BCA: Bicinchoninic acid assay
BMC: Bone mineral content
BMD: Bone mineral density
BP: Biological process (Gene Ontology)
BSA: Bovine serum albumin
BUN: Blood urea nitrogen
BV/TV: Bone volume fraction
Ca: Calcium
CC: Cellular component (Gene Ontology)
CCK-8: Cell Counting Kit-8
COL1A1: Collagen type I alpha 1 chain
COL1A2: Collagen type I alpha 2 chain
CPC: Cetylpyridinium chloride
Crea: Creatinine
CTX-I: Type I collagen cross-linked C-terminal telopeptide
DAB: 3,3’-Diaminobenzidine
DAPI: 4′,6-Diamidino-2-phenylindole
DCFH-DA: 2′,7’-Dichlorodihydrofluorescein diacetate
DMEM: Dulbecco’s Modified Eagle’s Medium
DXA: Dual-energy X-ray absorptiometry
E2: 17β-Estradiol
EDTA: Ethylenediaminetetraacetic acid
ELISA: Enzyme-linked immunosorbent assay
ESR1: Estrogen receptor alpha
FBS: Fetal bovine serum
FDR: False discovery rate
GO: Gene Ontology
GR: Glutathione reductase
GSH: Reduced glutathione
GSSG: Oxidized glutathione
H&E: Hematoxylin and eosin
HED: Human equivalent dose
HO-1: Heme oxygenase-1
HPLC: High-performance liquid chromatography
HRP: Horseradish peroxidase
IHC: Immunohistochemistry
IL-6: Interleukin-6
IL-10: Interleukin-10
IL-18: Interleukin-18
IL-1β: Interleukin-1 beta
IVIS: *In vivo* imaging system
KC: Keratinocyte chemoattractant (murine IL-8 homolog)
KEGG: Kyoto Encyclopedia of Genes and Genomes
LDA: Linear discriminant analysis
LEfSe: Linear discriminant analysis effect size
LPO: Lipid peroxidation
LPS: Lipopolysaccharide
MAR: Mineral apposition rate
mBMSCs: Mouse bone marrow mesenchymal stem cells
MDA: Malondialdehyde
MF: Molecular function (Gene Ontology)
ML385: Nrf2 inhibitor
MMA: Methyl methacrylate
MYC: Myelocytomatosis oncogene (transcription factor)
NC: Negative control
NOR: Normal control (sham-operated)
Nrf2: Nuclear factor erythroid 2-related factor 2 (NFE2L2)
NTA: Nanoparticle tracking analysis
NVs: Nanovesicles
Ob. N/BS: Osteoblast number per bone surface
Oc. N/BS: Osteoclast number per bone surface
OCN: Osteocalcin
OTU: Operational taxonomic unit
OVX: Ovariectomy / ovariectomized
PCoA: Principal coordinates analysis
PD_whole_tree: Phylogenetic diversity whole tree index
PFA: Paraformaldehyde
PMOP: Postmenopausal osteoporosis
PPI: Protein–protein interaction
PQs: *Panax quinquefolius*-derived nanovesicles
qPCR: Quantitative real-time polymerase chain reaction
RANKL: Receptor activator of nuclear factor kappa-B ligand
RM-ANOVA: Repeated-measures analysis of variance
RNA-seq: RNA sequencing
ROC: Receiver operating characteristic
ROI: Region of interest
ROS: Reactive oxygen species
Runx2: Runt-related transcription factor 2
s-Ca: Serum calcium
SCFAs: Short-chain fatty acids
SD: Standard deviation
SEM: Standard error of the mean
siNC: Negative control small interfering RNA
siRNA: Small interfering RNA
siNrf2: Nrf2-targeting small interfering RNA
SMI: Structure model index
SOD: Superoxide dismutase
Tb. N: Trabecular number
Tb. Sp: Trabecular separation
Tb. Th: Trabecular thickness
TCM: Traditional Chinese medicine
TEM: Transmission electron microscopy
TNF: Tumor necrosis factor
TRAP: Tartrate-resistant acid phosphatase
TRAP5b: Tartrate-resistant acid phosphatase isoform 5b
μCT: Micro-computed tomography
ZO-1: Zonula occludens-1
