# Supplementary material for: Panax quinquefolius-derived nanovesicles: a synergistic nanotherapy for postmenopausal osteoporosis via modulating gut microbiota, MYC-mediated inflammation, and Nrf2/HO-1 antioxidant pathway
Source: Front Microbiol. 2026 Jul 22;17:1836521. doi: 10.3389/fmicb.2026.1836521 (PMC13440151; doi:10.3389/fmicb.2026.1836521)
Supplement: Supplementary file 1 [file Supplementary_file_1.docx]

| Supplementary Table S1. Molecular docking results of naringin with core target proteins | | | | | |
| --- | --- | --- | --- | --- | --- |
| Target | PDB/ChEMBL ID | Binding free energy (kcal/mol, mean ± SD) | Cavity volume (Å³) | Key interacting residues | Interaction type |
| TNF | ChEMBL: CHEMBL224 | −8.5 ± 0.2 | 486 | Leu57, Tyr59, Ser60 | Hydrogen bond, hydrophobic |
| Nrf2 (NFE2L2) | ChEMBL: CHEMBL1293237 | −11.8 ± 0.3 | 712 | Arg415, Ser508, Gly367 | Hydrogen bond, π-π stacking |
| MYC | ChEMBL: CHEMBL1293229 | −9.6 ± 0.2 | 598 | Asn285, Gln352, Ile301 | Hydrophobic, van der Waals |
| ESR1 | ChEMBL: CHEMBL1485 | −8.9 ± 0.2 | 423 | Glu353, Arg394, Leu525 | Hydrogen bond, hydrophobic |
| IL10 | ChEMBL: CHEMBL1743125 | −8.9 ± 0.1 | 355 | Asp62, Phe65, Tyr69 | Hydrogen bond |

| Supplementary Table S2. qPCR analysis of key target genes in RAW264.7 cells and bone tissue | | | |  |
| --- | --- | --- | --- | --- |
|  |  |  |  |  |
| Gene | Group | RAW264.7 cells (relative mRNA expression, mean ± SD, n=3) | Bone tissue (relative mRNA expression, mean ± SD, n=6) |  |
| Nrf2 | Normal | 1.00 ± 0.08 | 1.00 ± 0.10 |  |
|  | RANKL / OVX | 0.42 ± 0.05*** | 0.38 ± 0.06*** |  |
|  | RANKL / OVX + PQs | 1.18 ± 0.12### | 0.95 ± 0.09### |  |
| HO-1 | Normal | 1.00 ± 0.09 | 1.00 ± 0.11 |  |
|  | RANKL / OVX | 0.35 ± 0.04*** | 0.32 ± 0.05*** |  |
|  | RANKL / OVX + PQs | 1.12 ± 0.10### | 0.88 ± 0.08### |  |
| MYC | Normal | 1.00 ± 0.07 | 1.00 ± 0.09 |  |
|  | RANKL / OVX | 3.45 ± 0.32*** | 3.12 ± 0.28*** |  |
|  | RANKL / OVX + PQs | 1.55 ± 0.15### | 1.42 ± 0.13### |  |
| IL-6 | Normal | 1.00 ± 0.10 | 1.00 ± 0.12 |  |
|  | RANKL / OVX | 4.21 ± 0.45*** | 3.85 ± 0.35*** |  |
|  | RANKL / OVX + PQs | 1.35 ± 0.12### | 1.28 ± 0.11### |  |
| Caspase-1 | Normal | 1.00 ± 0.08 | 1.00 ± 0.09 |  |
|  | RANKL / OVX | 3.12 ± 0.28*** | 2.95 ± 0.25*** |  |
|  | RANKL / OVX + PQs | 1.18 ± 0.10### | 1.12 ± 0.10### |  |
| Data are presented as mean ± SD. For RAW264.7 cells, “Normal” refers to untreated cells; “RANKL” refers to cells stimulated with 50 ng/mL RANKL; “RANKL + PQs” refers to RANKL-stimulated cells treated with 25 μg/mL PQs. For bone tissue, “Normal” refers to sham-operated mice; “OVX” refers to ovariectomized mice; “OVX + PQs” refers to OVX mice treated with PQs-H (30 × 10¹⁰ particles/kg). ***P < 0.001 vs. Normal group; ###P < 0.001 vs. RANKL or OVX group (one-way ANOVA with Tukey’s post-hoc test). Gapdh was used as the internal control. Relative expression was calculated using the 2^−ΔΔCT method | | | |  |
|  |  |  |  |  |
|  |  |  |  |  |
|  |  |  |  |  |
|  |  |  |  |  |
|  |  |  |  |  |
|  |  |  |  |  |
|  |  |  |  |  |
|  |  |  |  |  |

| Supplementary Table S3. Correlation analysis between serum markers and μCT parameters | | |  |
| --- | --- | --- | --- |
|  |  |  |  |
| Comparison | Correlation coefficient (r) | P value |  |
| Serum OCN vs. BV/TV | 0.82 | <0.001 |  |
| Serum CTX‑I vs. Oc.N/BS | 0.79 | <0.001 |  |
| Serum ALP vs. MAR | 0.68 | <0.01 |  |
